# Whole Plant Extracts for Neurocognitive Disorders: A Narrative Review of Neuropsychological and Preclinical Studies

**DOI:** 10.3390/nu16183156

**Published:** 2024-09-18

**Authors:** Alessandro Piva, Giulia Benvegnù, Stefano Negri, Mauro Commisso, Sofia Ceccato, Linda Avesani, Flavia Guzzo, Cristiano Chiamulera

**Affiliations:** 1 Department of Diagnostics and Public Health, University of Verona, 37134 Verona, Italy; alessandro.piva@univr.it (A.P.); giulia.benvegnu@univr.it (G.B.); cristiano.chiamulera@univr.it (C.C.); 2 NBFC, National Biodiversity Future Center, 90133 Palermo, Italy; stefano.negri@univr.it (S.N.); mauro.commisso@univr.it (M.C.); linda.avesani@univr.it (L.A.); flavia.guzzo@univr.it (F.G.); 3 Department of Biotechnology, University of Verona, 37134 Verona, Italy

**Keywords:** plant extracts, bioactive phytochemicals, rodents, humans, neuropsychological tests, cognitive functions

## Abstract

The incidence of neurodegenerative disorders like Alzheimer’s or Parkinson’s Disease, characterized by a progressive cognitive decline, is rising worldwide. Despite the considerable efforts to unveil the neuropsychological bases of these diseases, there is still an unmet medical need for effective therapies against cognitive deficits. In recent years, increasing laboratory evidence indicates the potential of phytotherapy as an integrative aid to improve cognitive functions. In this review, we describe the data of plant whole extracts or single compounds’ efficacy on validated preclinical models and neuropsychological tests, aiming to correlate brain mechanisms underlying rodent behavioral responses to human findings. After a search of the literature, the overview was limited to the following plants: *Dioscorea batatas*, *Ginkgo biloba*, *Melissa officinalis*, *Nigella sativa*, *Olea europaea*, *Panax ginseng*, *Punica granatum*, and *Vitis vinifera*. Results showed significant improvements in different cognitive functions, such as learning and memory or visuospatial abilities, in both humans and rodents. However, despite promising laboratory evidence, clinical translation has been dampened by a limited pharmacological characterization of the single bioactive components of the herbal products. Depicting the contribution of the single phytochemicals to the phytocomplex’s pharmacological efficacy could enable the comprehension of their potential synergistic activity, leading to phytotherapy inclusion in the existing therapeutic package against cognitive decline.

## 1. Introduction

The problem of cognitive deficits in several disorders, primarily in specific mental disorders but also in aging, is increasing as the average age increases. Globally, for example, dementia will affect 139 million people by 2050, compared to 55 million currently [1]. Because of the differences in impaired processes and functions, as mediated by distributed brain processes and neurochemical mechanisms, preventing cognitive decline requires precise diagnosis [2]. At the diagnostic—but also experimental—level, the cognitive performance is assessed using specific neuropsychological tests [3]. At the experimental level, valid animal models have been developed and recommended for translational value in order to identify potential drug targets [4,5]. Although there is an ongoing cross-validation approach between human and lab animal research aiming to identify novel pharmacological interventions for cognitive disorders, there is still an unmet medical need to improve efficacy, tolerability, and safety [6]. Moreover, non-pharmacological aids associated with pharmacotherapy may provide further improvement to integrative approaches, whether preventive, slowing the decline, or reversing the deficits.

Phytotherapies may offer potential aid in such an integrative approach. In the last decade, several herbal remedies and/or plant-active molecules have been proposed as nootropics, with the scope to have an alternative, or better, an adjunct, to synthetic drugs (for recent reviews, see [7,8]). Recent research approaches based on -omics methodologies suggest a novel strategy, which consists of testing the potential effects of whole plant extracts in toto in order to exploit a synergistic action on mechanisms underlying cognitive functions [9,10]. Due to their sessile nature, plants display an unmatched chemical diversity comprising a wide array of compounds that, either as a result of their specific coevolution with animals or just by coincidental interaction with organic targets, can exert various bioactivities in different organisms. Therefore, plants and plant extracts have been shown to act on one or more of the six neurocognitive domains defined by the Diagnostic and Statistical Manual of Mental Disorders (DSM-5; [2]): (1) perceptual-motor functions; (2) language; (3) learning and memory processes; (4) social cognition; (5) attentional processes; (6) executive functions [11]. These functions are assessed both in healthy subjects and in patients for diagnosis, disease progression, and rehabilitation monitoring of pathologies that show a progressive cognitive decline, for example Alzheimer’s Disease (AD), Parkinson’s Disease (PD), Huntington’s Disease (HD) or Amyotrophic Lateral Sclerosis (ALS), Mild Cognitive disorders [12]. However, even in the case of nootropics, the current evidence is limited and, in many cases, inconsistent, and the main limitation lies in the difficulty of developing and analyzing cognitive tests on preclinical models that can be positively translated into clinical tests [7,13].

With this narrative review, we aim to describe and compare efficacy data by plants and/or plant extracts obtained from neuropsychological as well as preclinical laboratory animal tests. We will describe recent findings on the effects of specific plant extracts or bioactive compounds on cognitive functions, as experimentally tested in human neuropsychological tests and studied in valid animal models, with final recommendations on the need for additional validation, translation, and modality-of-use studies in patients.

## 2. Materials and Methods

### 2.1. Search Criteria for Human Literature

A systematic search of the PubMed/MEDLINE database (https://pubmed.ncbi.nlm.nih.gov/, first accessed on 23 March 2023; last accessed on 30 October 2023) was conducted, using the following research string: plant AND human AND cognit* AND neuropsycholog* AND experiment*. The following filters were applied: humans (species), abstract, full text (text availability). The search was conducted in all fields.

The research yielded 33 records that were evaluated for inclusion based on the following criteria: (1) whole plant extracts or single active compound must be used, but not a mixture of different plants; (2) only legal plant species must be used; (3) it must be an experimental study; (4) it must be on humans; (5) it must target cognitive functions; (6) it must use neuropsychological tests.

The first selection was made based on the title and abstract. This selection resulted in the exclusion of 15 records because they were irrelevant (i.e., they were not papers on plant extracts, but the term “plant” appeared in the text used as a verb or was the last name of one of the authors). Further filtering was performed by reading each full text of the remaining documents to verify their inclusion in the final selection. After full-text screening, six articles were excluded: 1 did not use the whole plant extract, 1 used a combination of two plant extracts, and four did not use a plant species legal in the country of the study. Two additional articles were removed because they described studies still in progress, and the results still needed to be reported. Of the final 10 articles, three described the effects of Ginkgo biloba, one of *Melissa officinalis*, one of *Panax ginseng*, one of *Nigella sativa*, one of *Olea europaea*, one of *Vitis vinifera*, one of *Punica granatum* and one of *Dioscorea batatas*. All included studies were randomized and included adequate descriptions of the sample, inclusion and exclusion criteria, procedures, and statistical analysis.

**Figure 1:**
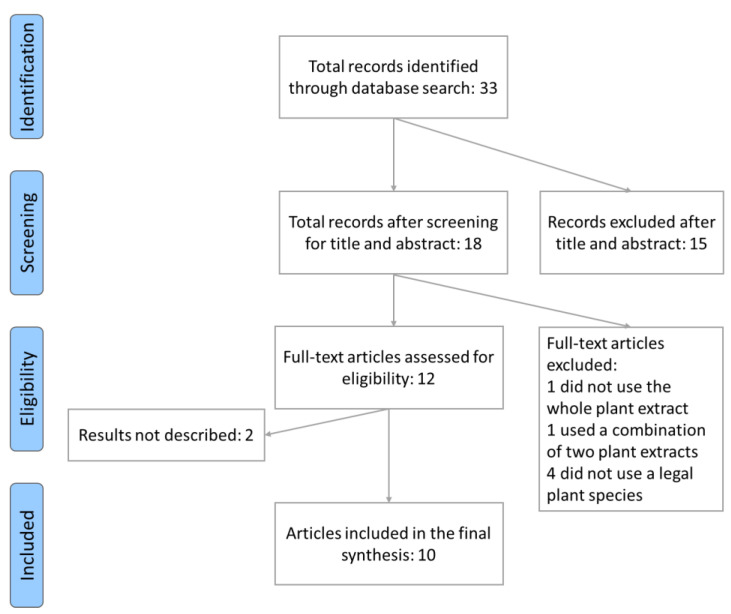
Flow diagram of the human search strategy. Adapted from [14].

The flowchart in Figure 1 shows the steps of the process.

### 2.2. Search Criteria for Preclinical Literature

A systematic search of the PubMed/MEDLINE database (https://pubmed.ncbi.nlm.nih.gov; first access on 13 April 2023; last access on 30 October 2023) was conducted, using the following research string: “name of the plant” AND cogni* AND (“mouse” OR “mice” OR “rat” OR “rats”) NOT review. For the name of the plant term, our searches were focused only on the plants that resulted from the search for the human literature, i.e., *Dioscorea batatas*, Ginkgo biloba, *Melissa officinalis*, *Nigella sativa*, *Olea europaea*, *Panax ginseng*, *Punica granatum*, and *Vitis vinifera*.

The following criteria were evaluated for records inclusion: (1) use of plant extracts or single active compound, but not a mixture of different plants; (2) use of preclinical animal models; (3) analysis of cognitive functions with validated behavioral tests. The research yielded a total of 215 records.

The first screening was made based on the title and abstract. This selection led to the exclusion of 10 records because (i) two were reviews, (ii) five were on humans, (iii) two were only in Russian, and (iv) one was only an abstract. Further filtering was performed by reading the Material & Methods and the Results sections of each paper, leading to the exclusion of 67 records because of the absence of cognitive experiments or the use of only plant mix. Finally, three and two reports, respectively, cited in articles on PG and NS were found in their bibliography and thus added to the review. The total number of manuscripts analyzed was 143, 1 for *Dioscorea batatas*, 66 for Ginkgo biloba, 4 for *Melissa officinalis*, 14 for *Nigella sativa*, 2 for *Olea europaea*, 45 for *Panax ginseng*, 5 for *Punica granatum* and 6 for *Vitis vinifera*.

**Figure 2:**
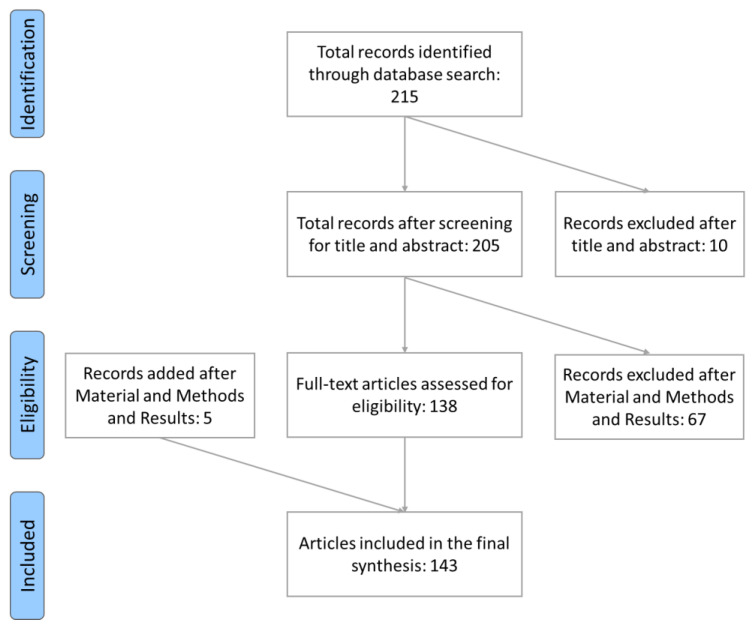
Flowchart diagram of the preclinical search strategy. Adapted from [14].

The flowchart in Figure 2 shows the steps of the process.

## 3. Results

### 3.1. Evidence of Plant Extract Effects on Human Cognition

Ten papers published between 2000 and 2021 met the inclusion criteria for the review, with large methodological heterogeneity arising from study design and duration, variability in sample characteristics, choice of cognitive domains explored, and neuropsychological tests used. The results are presented by grouping the studies that used extracts from the same plant species. Finally, because the review focuses on cognitive outcomes of plant extracts, other types of data (e.g., neuroimaging correlates, biochemical markers) that were present in some of the selected articles considered were not described.

Table 1 displays the characteristics and the main findings of the studies included in the review.

#### 3.1.1. *Dioscorea batatas*

A commercially available diosgenin-rich yam extract of *Dioscorea batatas* (DB) tuber (yam) was tested in a double-blind crossover Randomized Controlled Trial (RCT) by Tohda and colleagues [15].

Healthy volunteers were randomly allocated into two groups: one group (*n* = 17) was given 50 mg/day (d) of the extract of DB for 12 weeks (wk) and then received a placebo for another 12 wk; the other group (*n* = 11) received the placebo for 12 wk and then the extract of DB. A 6-wk washout period separated the two crossover intake periods. The Repeatable Battery for the Assessment of Neuropsychological Status (RBANS), an instrument that assesses five cognitive domains (immediate memory, visuospatial/constructional abilities, language, attention, and delayed memory), was administered as the primary outcome measure, while the Mini-Mental State Examination (MMSE) was used as a secondary measure. Diosgenin-rich yam extract consumption yielded significant increases in total RBANS score. This increase was independent of sex but was age-dependent: participants in the 47–81 age group significantly increased scores compared to placebo. Considering individual cognitive subtests of RBANS, participants treated with the diosgenin-rich yam extract improved their semantic fluency, a subtest of the language domain.

This suggests that in elderly subjects, intake of DB extract results in cognitive improvement with a major positive effect on language.

#### 3.1.2. *Ginkgo biloba*

The extract of Ginkgo biloba (leaves GB) has been used in three double-blind RCT studies. In the report by Mix and Crews and in one of the experiments described in the work of Elsabagh et al., participants were administered daily for 6 wk, respectively, with a dose of 180 and 120 mg/d, while in a third study by Santos et al., authors used a lower dose (80 mg/d) for 8 months (mth) [16,17,18]. Specifically, the study by Santos et al. on healthy elderly revealed improvements in the group treated for 8 mth with a daily dose of GB (*n* = 23) in several cognitive areas: visuospatial abilities (assessed by Block Design and Object Assembly of the Wechsler Adult Intelligence Scale-Revised -WAIS-R- and Corsi’s blocks), sustained and selective attention (measured with Digit Symbol of the WAIS-R and the Toulouse Pieron Concentrated Attention), working memory (assessed by Mental Control and Verbal Paired Associates from the Wechsler Memory Scale-Revised -WMS-R-) and information processing speed (assessed by timed tests). In addition, improvement was observed also in language and in the so-called general intelligence (as measured by the Comprehension, Vocabulary, and Similarities tests from the WAIS-R) [18]. In contrast, the work by Mix and Crews on 48 healthy elderly participants revealed improved executive functions, as measured by the Stroop Color and Word test, in the group treated for 6 wk (*n* = 24) compared to the control group [16]. In the latter report, authors also used the WMS-R, but no significant differences emerged. This result was possibly due to the subtests included (such as Logical Memory subtests), which did not show any significant results also in the work by Santos et al. (unlike the other WMS-R subtests used) [16,18].

Finally, in the article by Elsabagh and colleagues, the authors showed no significant effects by the 6-wk chronic treatment (total sample size = 40, experimental group = 20) but a significant effect by acute GB administration (total sample size = 52, experimental group = 26). In fact, a single 120 mg dose on young students significantly improved performance in sustained attention, long-term memory, and working memory, tested with the Paced Auditory Serial Addition Test (PASAT) and pattern-recognition memory task (PRM), both subtests of the Cambridge Neuropsychological Test Automated Battery (CANTAB) [17].

In conclusion, GB has been shown to improve memory and attention [17,18] and secondarily to have an effect on visuospatial abilities, information processing speed, intelligence, language [18] and executive functions [16].

#### 3.1.3. *Melissa officinalis*

In the double-blind balanced crossover RCT by Kennedy and colleagues, 18 healthy students received a placebo, a 300 mg dose, and a 600 mg dose of *Melissa officinalis* (leaves MO) separated by a 7 d washout period. After ingestion of 300 mg dose compared to placebo, a significant increase in accuracy and (marginally) speed of resolution of mathematical tasks in the Defined Intensity Stressor Simulation (DISS) battery was observed [19], suggesting an effect of MO on mathematical processing.

#### 3.1.4. *Nigella sativa*

In the double-blind RCT by Bin Sayeed et al. on 40 healthy elderly volunteers, authors tested the effects of 500 mg *Nigella sativa* (NS) administered two times per day (2/d) for 9 wk. The NS-treated group (*n* = 20) showed at the end of 9 wk an improvement over the control group in working memory (as measured by Logical Memory I of the WMS-R, Digit Span Test of the WAIS-R), long-term memory (LTM, assessed by Logical Memory II of the WMS-R), visuospatial abilities (measured by Rey-Osterrieth complex figure test), visual discrimination and selective attention (evaluated with Letter Cancellation Test and Trail making test-A and test-B) and executive functions (measured by Stroop Color and Word test) [20].

Therefore, NS appears to improve several cognitive functions, including memory, attention, executive functions, and visuospatial abilities.

#### 3.1.5. *Olea europaea*

The extract of *Olea europaea* (OE) was used in an open-label RCT to test the effects of extra virgin olive oil on healthy elderly when given for one year at a dose of 20–30 g/d [21]. The experimental group (*n* = 55) was assigned to a dietary regimen that included a Mediterranean diet in which every oil, including olive oil, high-oleic safflower oil, high-oleic sunflower oil, canola oil, and hydrogenated vegetable oils, was replaced by extra virgin olive oil. The control group (*n* = 55) followed the same Mediterranean diet but without oil substitution. Participants’ cognitive functioning was tested at baseline and after one year using the MMSE and a subscale of the Alzheimer’s Disease Assessment Scale -ADAS- assessing memory, praxis, language, and orientation (ADAS-cog). The results showed a greater reduction in ADAS-cog scores, i.e., improved test, after one year in the experimental vs. control group.

Noteworthy, the study reported only the total score of the MMSE and of the ADAS-cog and not the results of individual subtests, making it difficult to identify the effects of OE on specific cognitive functions.

#### 3.1.6. *Panax ginseng*

In a Prospective Open-label RCT by Lee et al., the effects of *Panax ginseng* (PG) roots extract were tested on the cognitive performance of Alzheimer’s disease patients (total sample size = 107) [22]. The experimental group (*n* = 59) was treated with 4.5 g/d of PG powder for 12 wk. Participants’ cognitive performance was monitored using the MMSE and the ADAS administered at baseline, at 4 wk and 12 wk after the treatment, and at 12 wk after PG withdrawal. An additional group (*n* = 9) received a higher dose of PG (9 g/d) to evaluate a possible dose-response effect. Results showed improvement over the control group in MMSE after 12 wk and in ADAS-cog at 4 wk and at 12 wk. No effects were observed after the 9 g/d dose of PG.

Again, as previously reported on [21] with OE, it is complex to identify the specific cognitive functions affected by PG, as only the total scores, but not the subtest scores, were reported for MMSE and ADAS subscales.

#### 3.1.7. *Punica granatum*

In the study of Petrou et al., a double-blind crossover RCT investigated the effect of *Punica granatum* (PuG) seed oil on 30 patients with multiple sclerosis [23]. Participants were divided into two groups: Group A (*n* = 15) was given a nano-formulation of pomegranate seed oil (GranaGard, 125 mg capsule) every day for the first 3 mth, and placebo for the following 3 mth; Group B (*n* = 15) was given a placebo for the first 3 mth, then GranaGard for the following 3 mth. Then, the two groups received GranaGard for an additional 6 mth. Participants were tested at baseline, after 3, and after 6 mth with a series of neuropsychological batteries and tests (See Table 1). Group A showed a significant improvement in verbal long-term memory and working memory (measured with CVLT-II) at 3 mth measurements compared with baseline. In contrast, group B showed no significant differences.

In light of this, PuG shows promising effects on memory in patients with multiple sclerosis, but further studies with larger samples are needed to confirm these findings.

#### 3.1.8. *Vitis vinifera*

In a prospective, double-blind RCT, Lee et al. explored the impact of grape consumption on cognitive decline in elderly subjects (total sample size = 10) [24]. The experimental group (*n* = 5) took 36 g of *Vitis vinifera* fruit (VV) extract twice a day for 6 mth. All participants were administered a series of batteries and neuropsychological tests (e.g., MMSE, WAIS-III, Rey-Osterrieth Complex Figure Test copy and delayed, Boston Naming Test—see Table 1) at baseline and after 6 mth to check for any effects on various cognitive functions including memory, language, executive functions, speed of information processing, visuospatial abilities, attention and working memory. There were no significant differences between the control and experimental groups in any of the tests used.

### 3.2. Evidence of Plant Extract Effects on Rodents Cognition

A considerable number of articles published between 1992 and 2023 accounted for preclinical evidence of plant extract effects on neurocognitive function. The effects of these extracts were investigated on different cognitive decline models, such as neurodegenerative transgenic or pharmacologically induced disease models, ischemic stroke-induced vascular dementia, and aging, on both male and female rodents. The effects of plants on other models, such as stress, are cited for reference. The results of behavioral testing are grouped below by plant species, regardless of the plant origin or extract source. Histological, molecular, and genetic results are not described; however, cognitive effects were associated with antioxidant, anti-inflammatory, and neuroprotective activity of the extracts by nearly all the reports. Herbal extracts were assumed to be orally (po) administered except when differently specified, while scopolamine (SCO) was always injected intraperitoneally (ip). The characteristics and the main findings of the studies included in the review, except for those only cited for reference, are reported in the Supplementary Table S1.

#### 3.2.1. *Dioscorea batatas*

In the same study reported for the human literature, the authors analyzed the effect of diosgenin-rich yam extract of *Dioscorea batatas* (DB), suspended in soybean oil, on normal female mice. After 7 d of administration at 0.259 mg/kg/d, the Novel Object Recognition test (NORT) was performed with an interval of 48 h between training and test. DB showed a significant enhancement of object recognition memory at the test compared to the training session [15].

#### 3.2.2. *Ginkgo biloba*

The extract of Ginkgo biloba (GB), mainly as the commercially available EGb761^®^ or as an equivalent extract standardized with a similar amount of flavonglucosides (~24%), terpenoid lactones (~6%) and ginkgolic acids (<5 ppm) [25] has been used in 59 of 67 papers. The other reports used either the main bioactive ingredient in GB leaf extracts, ginkgolide B [26,27,28], or the sesquiterpene lactone bilobalide [29,30].

The group of Tian and colleagues, in three consecutive reports, demonstrated a positive effect of GB on an experimentally induced model of Alzheimer’s Disease (AD). Specifically, they showed that EGb761 20 mg/kg/d for 20 d was able to ameliorate performances in the Morris Water Maze (MWM) task, i.e., to decrease the escape latency along the training days and to increase the number of target platform crossings and the % time of swimming in the target quadrant during the probe test, and thus to ameliorate spatial learning and memory in rats treated with aggregated Aβ25–35 bilaterally in hippocampus. The same treatment showed a higher effect when in combination with hyperbaric oxygen [31,32,33]. A similar effect on the MWM task was observed by Tu et al. after EGb761 20 mg/kg/d administration for 23 d in mice intracerebroventricularly (icv) injected with Aβ1–42 peptide [34]. To model AD, icv injection of the pancreatic toxin streptozotocin (STZ) was also used. Hoyer and colleagues bilaterally icv injected STZ at 0.25 mg/2 mL per site three times during the experiment, while EGb761 50 mg/d was available with a normal diet for 80 d. Here, EGb761 was able to partially recover working and reference memory in a holeboard test and to partially restore the acquisition and recall of aversive shock associative memory in a passive avoidance (PA) task [35]. On the other hand, transgenic (tg) mouse models of AD overexpressing the human APP and/or PSEN1 proteins were used in seven reports on GB effects on cognition. In particular, EGb761 50 to 70 mg/kg/d for 90 d to 6 mth, but not for 2 mth [36,37], significantly recovered the impaired spatial learning and memory functions in MWM and Barnes Maze Test (BMT) [36,37,38,39,40]. Positive effects on MWM and on NORT, as an enhanced preference for the novel vs. familiar object, were also observed for ip EGb761 at 20 or 30, but not 10 mg/kg/d, for 4 mth in 5xFAD mice [41]. Finally, ginkgolide B at 30 and 40 mg/kg for 3 wk recovered to normal levels the spatial learning and memory abilities sampled by MWM and NORT tasks in SAMP8 male mice, i.e., a senescence-accelerated mouse model with APP overproduction and early memory disturbances [28]. To induce AD-like symptoms, other authors used the hyperhomocysteinemia model. In one study, homocysteine (Hcy) was given at 400 μg/kg/d iv for 14 d. To mimic a preventive or curative treatment, EGb761 400 mg/kg/d for 7 d was given simultaneously or after Hcy, respectively. EGb761 enhanced MWM performance and STM and LTM in a fear conditioning task (FC) in both preventive and curative settings [42]. On the same model, induced by 1 g/kg methionine to female rats throughout pregnancy, the contemporary administration of EGb761 100 mg/kg 2/d did not revert spatial deficits of 75-d-old offspring in MWM [43]. In conclusion, considering AD models, GB has shown enhancing properties on spatial learning and memory, on recognition memory, and, even partially, on fear-association and on working and reference memory, when given for at least 20 d at doses lower or comparable to the clinical one [44]. Noteworthy, GB has also shown preventive and curative effects on short- and long-term memory when administered at very high doses for a short period. However, it did not prevent cognitive deficits in the offspring of AD-induced female rats.

A number of studies also investigated the effects of GB extracts on cognitive deficits associated with experimentally induced vascular dementia. A novel GB extract, consisting of EGb761 enriched with pinitol, intravenously (iv) administered for 14 d at the dose of 10.5 mg/kg to mice after transient middle cerebral artery occlusion (MCAO) improved spatial learning and memory as showed by MWM and NORT [45]. After a 2-vessel bilateral common carotid artery occlusion (2VO) surgery, a 20 mg/kg GB extract was administered for 21 d ameliorated spatial memory as shown by an 8-arm radial maze (8-ARM) test [46]. On the same model, EGb761 showed positive effects either for 2 wk before or ip for 1 mth after ischemia on spatial cognition at 100 mg/kg [47,48], a dose-dependent relief of response latency in PA test at 100, 200, and 400 mg/kg for 5 d before 2VO [49], and improved spatial learning and memory in MWM at 50 mg/kg for 15 d and for 1, 2, and 4 mth [50]. On the other hand, EGb761 to rats after a 4VO (i.e., 2VO with vertebral arteries cauterization) showed positive effects on acquisition but not on retention of spatial memory in aversive 8-ARM at 150 mg/kg 3 times/d (3/d) for 4 d [51], and no effects on MWM as acute 40 mg/kg [52]. Similar to EGb761, also ginkgolide B and bilobalide showed positive effects on 2VO rats’ cognition, respectively, as 1.0 mL of 1.0 mg/mL solution for 14 d ip on MWM and Y-maze [27] and at 2, 4, and 8 mg/kg for 60 d on MWM [29]. In vascular dementia models, chronic and subchronic GB treatment, even at low doses, showed positive effects on reference and working memory, spatial learning and memory, and fear-associative and recognition memory. The use of intravenous injections or of single bioactive components can reduce the dose necessary to evoke a cognitive improvement.

Aging as a cognitive decline model has been used since 1994. In fact, Rapin et al. showed that EGb761 50 and 100 mg/kg for 21 d before experimental sessions to both 4 mth and 20-mth-old rats was effective in recovering performances to control levels in an operant discrimination task when an auditory stressor was introduced during learning, thus indicating an increased behavioral adaptation despite negative environmental influences [53]. Stoll and colleagues reported that EGb761 100 mg/kg for 3 wk inhibited the short-term memory (STM), but not the LTM, decline of aged rats in PA [54]. In 1998, Winter observed significant positive effects in spatial learning and working memory as assayed by a delayed nonmatching to position (DNMTP) task in an 8-ARM in 20 and 26-mth-old rats daily lifetime treated with EGb761 100 and 200 mg/kg [55]. Lately, EGb761 60, but not 30, mg/kg ip for 30 d to young male rats was shown to increase spontaneous recognition in an olfactory STM test; similar results were also observed for acute 60 and 120 mg/kg ip to young male and for 60 mg/kg ip to aged female rats [56]. A similar chronic EGb761 treatment exhibited amelioration of spatial learning and memory at 60 and of spatial cognitive flexibility at 30 and 60 mg/kg in MWM in 74 to 78-wk-old male rats [57]. Conversely, 100 mg/kg EGb761 to 18-mth-old female rats showed only mild positive effects in MWM [58]. Finally, in two reports, aged mice were exposed to stressor agents to analyze the effect of EGb761 on such a combined model. Ward et al. did not observe any significant cognitive effects on EPM when EGb761 was given at 100 mg/kg for 82 d to 20-mth-old mice to prevent cognitive deficits after forced swimming stressful exposure [59]; on the other hand, Pardon et al. showed restorative effects for EGb761 50 mg/kg for 7 mth on 17–18 and 23–24-mth-old mice exposed to a chronic unpredictable mild stress protocol for 4 wk. In particular, the authors showed an improvement in decision-making skills in a T-maze task, as an increased information processing speed to younger littermates’ levels [60]. To conclude, acute and chronic administration of EGb761 relieved cognition of aged male and female animals, particularly on recognition, spatial, and working memory, as well as on fear and non-fear associative learning and memory. Contrasting results were shown for aged animals under stressful conditions.

In the previously mentioned work by Harada et al. [49], authors investigated the effects of EGb761 both on ischemic stroke- and scopolamine-induced memory impairments. While the treatment was effective against the former, SCO 0.5 or 1 mg/kg-induced deficits in PA were unaffected by EGb761, even at 400 mg/kg. In contrast, the earlier report by Chopin and Briley showed no effects on acquisition but a significant effect on memory retention test in PA after EGb761 150, 300, 400, and 500 mg/kg acute ip against SCO 1 mg/kg [61]. On the same test, the standardized GB extract Ginkgocer increased latency time at 30 and 60 mg/kg for 7 d vs. SCO 3 mg/kg [62]. Furthermore, ginkgo ketoester tablets, similar to EGb761 extract, 58.5 mg/kg for 15 d ameliorated spatial memory in MWM vs. SCO 3 mg/kg [63], while EGb761 8.27 mg/kg 3/d for 15 d enhanced training in MWM vs. 30 d-SCO 1 mg/kg [64]. On both these reports, GB showed higher effects in combination with donepezil, at 0.65 for the former and at 0.5 or 1 mg/kg for the latter. Recently, two more articles showed positive effects of GB extract: the first showed that 400 mg/kg EGb761 for 27 d vs. 1 mg/kg SCO attenuated impairment in MWM and the fear-motivated associative learning and memory deficits in FC [65], while the second showed that 40 mg/kg EGb761-like GB leaf extract for 7 d vs. acute 1.2 mg/kg SCO improved spatial memory in NORT and in Y-maze [66]. In summary, chronic and subchronic EGb761—and EGb761-like—dosing has shown improving effects vs. scopolamine on spatial, recognition, and fear learning and memory, and on short-term working memory, even at low doses. However, differently from simple fear conditioning, GB extract effects on a more complex fear-motivated behavior showed contrasting evidence, even at very high doses.

As for scopolamine, also D-galactose (D-gal) with or without combined aluminum chloride injection (AlCl_3_) has been used to induce a cognitive decline in rodents. In Wang et al., rats were ip treated simultaneously for 8 wk with D-gal 100 mg/kg and with 0.875, 1.75, or 3.5 mg/kg EGb761-like GB extract. Cognitive abilities evaluated every 2 wk with Y-maze showed an amelioration of spatial working learning and memory from the sixth week for the medium and higher doses but not for the lower ones [67]. Similarly, simultaneous daily treatment with D-gal 0.5% 10 mL/kg and with 8.75, 17.5, or 35 mg/kg EGb761 for 6 wk improved MWM at training and test [68]. Finally, Liu et al. showed that ginkgolide B 0.1% for 4 wk on with D-gal + AlCl_3_ treated mice recovered deficits in NORT [26]. To conclude, GB extract improved recognition and working memory, as well as spatial learning and memory, when given against cognitive deficits induced by D-galactose alone or in combination with aluminum chloride.

Finally, on Parkinson’s Disease (PD) models of cognitive decline, two reports have been published. Firstly, 1-methyl-4-phenyl-1,2,3,6-tetrahydropyridine (MPTP) 30 mg/kg/d for 5 d was administered ip to selectively destroy the nigrostriatal dopaminergic neurons. Thereafter, mice were treated for 14 d with EGb761 or G. biloba dropping pill (GBDP), a unique Chinese preparation of GB leaf extract—different from EGb761—at 50 mg/kg. Here, the authors demonstrated a significant improvement by GBDP, but not EGb761, in MWM performance [69]. Secondly, the preventive or curative effects of EGb761-like extracts were investigated in a rotenone-induced PD-like disease. For the preventive effects, the EGb761-like extract was administered at 20 mg/kg for 11 d before a 10 d coadministration with rotenone (ROT) 1.5 mg/kg ip; for the curative effects, ROT was administered for 11 d before a 10 d coadministration with the EGb761-like extract. The extract showed preventive but not curative effects in Y-maze [70]. Thus, for PD models, results are unclear due to few and contrasting evidence on EGb761 efficacy on spatial learning and memory.

GB leaf extracts were shown to be effective on cognitive functions also in a number of other models, such as on stress [30,71,72,73,74,75,76], on epilepsy [77,78] on trimethyltin—[79,80], bisphenol A—[81], or chronic fluorosis-induced cognitive impairment [82], on collagenase-induced hemorrhagic stroke [83], against serotonin 1A receptors (5-HT1ARs) antagonism [84], against alcohol and dopamine D1Rs antagonism [85], on yohimbine [86] or cannabis-induced memory impairments [87], on naïve rats [88,89], on positive Gz acceleration model [90], on a tg diabetes model [91], and against hypobaric hypoxia [92].

#### 3.2.3. *Melissa officinalis*

The extract of *Melissa officinalis* leaves (MO) has been tested for its influences on cognitive functions and scopolamine-induced deficits. In 2016, Ozarowski et al. treated male rats with 200 mg/kg MO for 28 d. On the last day, acute 0.5 mg/kg SCO was injected 30 min after MO. Cognitive functions analysis showed that MO had no effects on STM in NORT, while a significant effect was observed on LTM in PA but abated by SCO [93]. Thus, MO showed a positive effect on long-term fear memory per sé but not on scopolamine-induced deficits. MO extract showed positive effects on cognitive functions also after chronic stress [94], epilepsy [95], and a diabetes model [96].

#### 3.2.4. *Nigella sativa*

The pathological models investigated for *Nigella sativa* (NS)-related cognitive recovery were D-gal, ischemic stroke, scopolamine, and AD models. The first model was induced by D-gal + AlCl_3_ 60 + 10 mg/kg/d for 6 wk, with the last 2 wk concomitantly to thymoquinone (TQ), i.e., the main active constituent of NS seed extract oil, at 10, 20 and 40 mg/kg for 14 d. PA tests at 24 h and 48 h after learning sessions revealed a positive effect of TQ, mainly at 20 mg/kg, on the learning and memory ability of rats [97]. This latter dose also produced restoring effects on MWM in the same model [98]. Ischemic stroke was induced by the 2VO protocol in two different studies. In the first, NS MetOH seed extract oil 1 mL/kg was given for 10 d before and for 70 d following 2VO. NS-treated group showed improvement in working memory, as well as in spatial STM and LTM vs. model in MWM [99]. On the same task, NS extract at 200 and 400 or TQ at 20 and 40 mg/kg ip for 10 d—3 d before and 7 d after 2VO—which demonstrated improvement of spatial learning and memory [100]. In the report by Abdelghany et al., SCO-induced impairment was reverted by different herbal extracts administered 90 min after 2.2 mg/kg SCO for 2 mth: NS 40 mg/kg, Rosmarinus officinalis 200 mg/kg, Salvia officinalis 600 mg/kg and PG 200 mg/kg—the latter was not found for the PG literature. All the herbal extracts showed spatial learning and memory enhancement vs. SCO in NORT, Y-maze, and MWM [101]. Finally, TQ at 5 and 10 mg/kg ip for 4 wk recovered PA associative learning in Aβ1–42-treated rats [102]. In summary, NS seed extract or its main constituent TQ was able to recover deficits in spatial learning and memory and in fear associative behavior when given to D-gal, ischemic stroke, and AD models, and also in recognition and working memory against scopolamine-induced cognitive decline.

NS extract or TQ showed positive effects on cognition also against epilepsy [103,104], glutamate toxicity [105], Cannabis sativa-induced moto-cognitive dysfunction [106], environmental pollutant [107], chemotherapy-induced cognitive impairment [108], on naïve rats [109], and on naïve rats when chronically treated during youth [110].

#### 3.2.5. *Olea europaea*

*Olea europaea* (OE) effects on cognition have been studied twice: the first report showed positive effects on learning and memory of OE (and NS) in naïve rats [109], while the second showed a reduction of fear conditioning in a streptozotocin-induced diabetes model [111].

#### 3.2.6. *Panax ginseng*

*Panax ginseng* (PG) extracts were obtained from roots in two different preparations: white (or unspecified) PG or red PG (RG). The difference between the two relies on steaming: while white PG roots are used after washing and drying fresh material, RG is obtained following washing, steaming, and drying steps. The most important aspect of this process is the increased concentration of heat-converted ginsenosides in red ginseng transformed from naturally occurring ginsenosides of the fresh roots [112].

SCO-induced deficits in cognition have been the most investigated model for PG. In their work in 1995, Nitta and colleagues used 8-ARM to analyze the effect of acute PG ethanol (EtOH) extract or its lipid- or water-soluble fraction (WSF) on rats’ cognition after 0.3 mg/kg SCO, showing that only the whole extract and the WSF restored spatial memory. Moreover, the authors declared that they had previously demonstrated PG extract efficacy on middle-aged rats in MWM and against SCO alterations in T-maze on rats [113]. Similarly, in 2016, the water-soluble oligosaccharides of PG extract at 40 and 80 mg/kg ip recovered spatial learning and memory at training and test in MWM and NORT, respectively, after a 10- or 7-d treatment [114]. On the other hand, Hsieh et al. showed that a 7-d administration of 500 or 1000 mg/kg PG, as well as Panax notoginseng—so-called Chinese ginseng, similar in chemical composition to PG, but with different concentrations of the same metabolites—MetOH extracts significantly preserved associative memory in PA after 1 mg/kg SCO [115]. Spatial learning and memory in MWM were also preserved by PG EtOH extract at both 100 and 200 mg/kg for 14 d after acute 2 mg/kg SCO [116]. Red ginseng (RG), as well as its hydrolyzed form (HRG) enriched in compound K—a secondary ginsenoside biotransformed from major ginsenosides—demonstrated restorative effects against SCO. In particular, RG 300 or HRG 50, 100, or 300 mg/kg for 10 d before 1 mg/kg SCO restored associative learning impairment and spatial learning and memory ability in Y-maze, PA, and MWM on nuclear factor E2-related factor 2-wild-type (Nrf2-WT), but not on Nrf2-KO mice, suggesting the involvement of Nrf2 signaling in RG-induced learning and memory enhancement [117]. Even different cultivation procedures influenced PG’s effects on cognition. A study on extracts from PG cultivated under (i) normal conditions with white LED light, (ii) in a nutrient bath with white LED light, or (iii) under normal conditions but with white, blue, and red LED lights, i.e., smart farming conditions, all given to mice at 200 mg/kg in parallel to 2 mg/kg SCO, showed PG ginsenosides altered concentration and enhancing effects on NORT, Y-maze, and PA only when grown under smart farming conditions [118]. Finally, effects on MWM, NORT, PA, and Y-maze were also investigated for ginseol k-g3, i.e., a fraction enriched in Rg3 and for 20(S)-protopanaxatriol (PPT). The former was given throughout the behavioral analysis at 12.5, 25, 50, 100, and 200 mg/kg; on the same report, also RG 100 and Rg3 20 and 40 mg/kg were used. Significant effects were observed on PA for all the treatments and only for Rg3 40 and k-g3 50 and 200 mg/kg on MWM [119]. Similarly, ip PPT 20 and 40 μmol/kg for 27 d counteracted 9 d SCO 0.75 mg/kg impairment on spatial learning and memory in NORT and MWM and on nonspatial memory in PA [120]. In conclusion, PG as a whole extract or as a specifically enriched fraction, as well as single PG extract-derived bioactive components, showed amelioration in working and reference memory, recognition memory, spatial learning and memory, and fear associative behavior.

As much as SCO, D-gal with or without combined aluminum chloride injection (AlCl_3_) was used in eight reports. Only one investigated the effects on PG and RG MetOH extracts, given at 800 mg/kg for the last 7 wk of a 9-wk-long D-gal treatment at 500 mg/kg/d. While RG showed ameliorated search latency, reference, and working memory in 8-ARM, PG effects were limited to the latter function [121]. Two more papers showed a significant effect on MWM when Ginseng proteins were given 2/d at 50 or 100 mg/kg during the last 30 d of a 90 d-long D-gal + AlCl_3_ (60 + 40 kg/kg/d) treatment [122,123]. The Ginsenoside Rg1 20 mg/kg/d ip for 28 d during a 42 d-long 120 mg/kg/d D-gal sc improved spatial memory in MWM [124,125] as well in MWM and PA when given at 10 or 20 mg/kg ip for 20 d after D-gal + AlCl_3_ [126]. Positive effects on MWM were observed also for Rg2 10 and 20 mg/kg/d during the last 4 wk of a 8-wk-long 800 mg/kg ip D-gal [127] and for Rg3 20 mg/kg/d given simultaneously to D-gal 60 mg/kg/d ip for 60 d [128]. As for scopolamine experiments, ginseng extracts or single ginsenosides showed positive effects on spatial learning and memory and on fear-associative behavior against D-gal-induced deficits, even as a preventive or curative agent for Rg1. Note that, unlike PG, RG was effective in both reference and working memory.

Different protocols were used for AD model induction. As the tg model, the senescence-accelerated SAMP8 mouse model administered with a ginsenosides mixture at 100 and 200 mg/kg/d for 7 mth showed improvement of MWM learning and memory and a delayed associative memory decline in PA task [129]. As induced models, 500 μg of advanced glycation end products (AGE)—products of nonenzymatic reactions between sugars and proteins and lipids that can bind to AGE Rs (RAGEs), leading to AD-like changes –were injected bilaterally into the hippocampal CA3 areas of rats. Then, PG water extract was given at 250, 500, or 1000 mg/kg/d for 30 d and tested for cognitive functions, showing amelioration in PA and in MWM [130]. On the same tasks, positive effects were also shown by fermented ginseng berry extract at 100, 200, or 400 mg/kg/d for 6 wk after icv injection of the cholinotoxin AF64A [131]. PGL-1 fraction, containing glycoproteins with low molecular weight, at 40, 80 and 160 mg/kg/d ip for 35 d following Aβ25–35 bilateral injection in hippocampus prevented decline in MWM performance [132]; the same effect on MWM was observed after 6 wk of Rg2 at 25, 50 and 100 mg/kg/d on the same experimental model [133] and after Rg1 at 5, 10, and 20 mg/kg/d for 25 d following AD induction by icv okadaic acid—an inhibitor of the protein phosphatases PP1 and PP2A that are involved in the tau protein-hyperphosphorylation hallmark of AD [134]. Finally, Rg5 at 5, 10, and 20 mg/kg for 27 d dose-dependently enhanced memory retention in PA and spatial learning and memory in MWM when given after an icv injection of STZ [135]. In conclusion, PG extracts of different compositions or single active ginsenosides induced improvement of spatial learning and memory and of fear associative behavior, at both low and high doses, on genetic or induced models of AD.

The vascular dementia model was developed, like for GB, with either MCAO or 2VO. The former was used in 2014 on male rats, treated with Rd 30 mg/kg ip 1 h before MCAO and with 10 mg/kg/d for 28 d after MCAO. Rd improved cognitive function as shown by MWM and NORT tasks [136]. For the latter, two substances were used: Rd at 10 or 30 mg/kg/d ip for 21 d, inducing improvements in MWM [137], and 20(S)-protopanaxadiol 5, 10, and 20 mg/kg/d sc for 3 wk, ameliorating memory and learning performances in MWM and Y-maze [138]. A PG standardized extract was also used in a combined model, i.e., administered at 50 and 100 mg/kg for 8 wk after 2VO and MCAO, producing dose-dependent enhancement in MWM [139]. Thus, evidence of efficacy against vascular dementia was mainly from ginseng active metabolites, rather than PG extract, and showed positive effects on working and recognition memory and on spatial learning and memory.

Lastly, for the aging model, Nitta et al. gave PG EtOH extract at 8000 mg/kg/d to 22–27 mth old rats for 33 d before 8-ARM or for 12 d before operant discrimination trials. PG was effective on the former but not on the latter test [140]. More recently, a ginsenosides mixture at 0.028%, 0.056%, and 0.112% given to 12-mth-old female mice for 8 mth improved MWM and PA [141], and 3 mth of RG extract at 200 mg/kg/d to 21-mth-old mice ameliorated memory impairment in Y-maze, NORT, and MWM [142]. Even limited reports on ginseng efficacy against aging-related cognitive decline showed amelioration in working, recognition, and fear memory, as well as in spatial learning and memory.

Further positive effects on cognition were observed against stress [143,144,145,146,147,148], sleep deprivation [149,150], alcohol, dopamine and muscarinic Rs antagonism [85,151], diabetes-derived deficits [152], kainic acid- [153] or TMT- [154] or domoic acid-induced disfunctions [155], and pollutant PM2.5 exposure [156].

#### 3.2.7. *Punica granatum*

*Punica granatum* (PuG) has been used against different pathological conditions. On the AD model, the standardized and commercially available PuG extract POMELLA at 100 and 200 mg/kg/d given to 24–30-mth-old R1.40 APP tg mice for 3 wk failed to improve MWM and Y-maze performance [157]. The hydroethanolic extract of PuG peel was tested against SCO-induced deficits: 200, 400, and 800 mg/kg/d doses for 3 wk were able to ameliorate spatial learning and memory at training and test in MWM, as well as to increase the time spent in the light compartment and to decrease the time spent in and the frequency of entry in the dark chamber in PA vs. SCO 2 mg/kg ip administered during the last week [158]. Similarly, PuG EtOH seed extract at 250 and 500 mg/kg/d for 21 d to aged mice or to young mice treated with SCO showed recovered cognitive functions in PA, seen as increased step-down latency, and in EPM, seen as decreased transfer latency vs. control [159]. Thus, PuG extract has shown efficacy in spatial learning and memory, working memory, and fear-associative behavior in both AD and scopolamine models.

Finally, PuG flower extract powder ameliorated cognitive performances in a diabetes model [160], while a PuG extract including flesh and seeds was shown to enhance spatial learning and memory in a chronic opioid treatment model [161].

#### 3.2.8. *Vitis vinifera*

In the field of *Vitis vinifera* studies, five different compounds were used in six different reports. In the oldest one, the aqueous extract of VV seed was given at 100, 200, and 300 mg/kg/d for 15 d to naïve rats. VV dose-dependently accelerates the acquisition and increases the memory retention and recovery of an AA response vs. SCO 1 mg/kg ip [162]. Furthermore, SCO 0.8 mg/kg/d was given ip during the last week of a 6-wk-long icv dosing of vitisin A at 1 or 100 ng/μL—a resveratrol tetramer derived from VV stembark –. Here, both doses were restored to control levels of the spatial learning and memory abilities in Y-maze and PA tasks [163]. Using the same experimental protocol, the same research group led by Kim showed recovering effects on NORT and PA vs. SCO for ampelopsin A, another compound derived from VV stembark [164].

The AlCl_3_ model of AD was used to investigate the effects of VV fresh fruit-derived powder. 250 and 500 mg/kg/d of this extract were given for 16 wk after AlCl_3_ 100 mg/kg/d for 8 wk. In MWM, VV at both doses reversed deficits at acquisition and at test [165]. The same extract at 400 mg/kg/d along with AlCl_3_ 100 mg/kg for 45 d normalized PA performances vs. model [166]. On the same model, a polyphenolic extract of VV leaves at 100 mg/kg for 21 d ameliorated cognitive abilities in a T-maze task [167]. In summary, the VV extract from seeds, fruits, and leaves, or even active compounds from stembark extract, demonstrated positive modulation of spatial learning and memory, fear associative behavior and recognition, and working memory against scopolamine and AD models of cognitive decline.

## 4. Discussion

In this review, we selected and reported the experimental studies that described the effects of whole plant extracts, phytochemical mixtures, or specific phytochemicals on neurocognitive functions.

One of the criteria for selection was the type of assessment methodology, i.e., cognitive functions investigated with validated neurocognitive tasks [168]. One of the criteria for selection was the type of assessment methodology, i.e., cognitive functions investigated with validated neurocognitive tasks [168] (see Supplementary Materials S1 and S2 for a brief overview of the clinical tests and preclinical tasks accounted). Our review, therefore, describes the studies of a limited number of plants, just those that met this methodological search criterion. In spite of differences between some tasks across the reviewed studies, the findings could be grouped into standardized cognitive functions as defined in the international classifications [169].

Another aim of our review was to compare the findings described in humans to the large body of experimental data reported by preclinical non-human research. Thus, we focused our preclinical search on those plants initially selected from the human studies and compared clinical to preclinical laboratory animals’ evidence. The reason for this species-species comparison was two-fold: (i) the availability of animal models with translational value to the corresponding human tests, supporting predictive validity, and (ii) the possibility of correlating brain mechanisms underlying preclinical behavioral responses to human findings. Therefore, our approach allowed us to select and discuss experimental data about plant effects on specific neurocognitive functions under controlled conditions and, in the meantime, provide mechanistic rationale and/or hypothesis from translational-validated preclinical animal studies.

Noteworthy, studies on laboratory animals were performed with models of different CNS disorders that are known to affect cognitive functions, such as Alzheimer’s Disease or dementia models. Therefore, the species-species comparison in our review may suggest potential effects in humans, as inferred by the neurocognitive test batteries, along with potential therapeutic applications for cognitive dysfunction in specific CNS disorder patients, thanks to the data from animal translational models.

Another important finding from our analysis is the comparison between evidence where plants were administered as different types of extract, such as a whole extract, a specific plant part(s), single active compounds, or compound mixtures, with different dosing modalities, such as acute, subchronic, or chronic, and through different routes of administration. Although species differences, these data may, however, help to define commonalities of the dosing/plant constituents relationship informing future clinical trial protocols.

**Figure 3:**
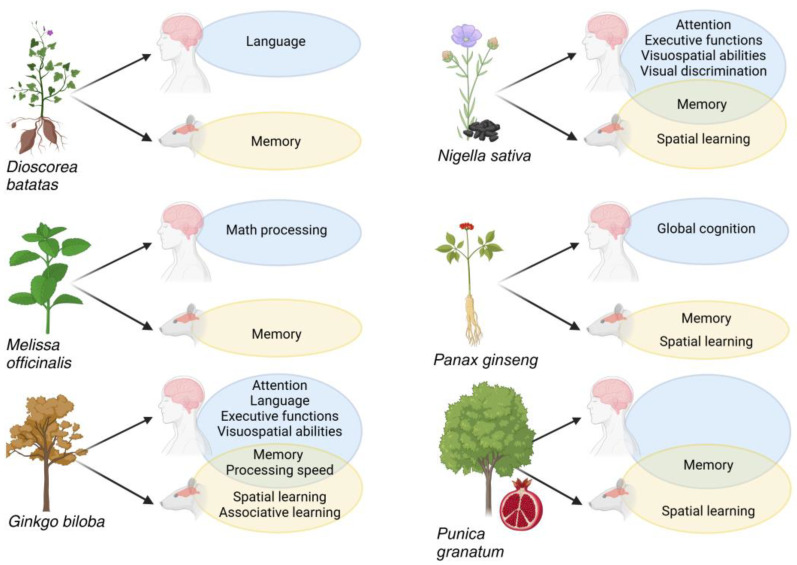
Schematic summary of the plant species showing more preclinical and clinical effects examined in the review. On the right of each plant species, the neurocognitive functions influenced by plant extract administration in humans (blue circle), in rodents (orange circle), or in both (circles overlap) are shown. Created with BioRender.com.

The comparison between human vs. animal lab studies showed some common, as well as different, effects between species (Figure 3). In other cases, we observed the lack of data at preclinical levels either for the lack of reports or for the kind of functions considered (e.g., language).

The plant with the greatest number of reported cognitive effects was *Ginkgo biloba*. This plant has been known for a long time for its different therapeutic effects, including the amelioration of cognitive performance in Alzheimer’s Disease (AD) [170] as reported anecdotally and from observational and interventional, including randomized, studies [171,172,173,174]. Regarding the focus of our search, we found that G. biloba improved task performance in attention, language, executive functions, and visuospatial abilities tests (Table 1). Moreover, it improved memory and processing speed, effects that have also been observed in animal studies (Table 1 and Supplementary Table S1). In laboratory animals, G. biloba also improved spatial and associative learning, with the latter as a process that could facilitate other types of memory performance [175,176].

The similarity between human and preclinical findings was also observed for *Nigella sativa*, with similar effects on memory performance. N. sativa effects in humans were like those described for G. biloba, i.e., an improvement of performance in attention, language, executive functions, and visuospatial abilities tasks (Table 1). The plant was also able to improve spatial learning in rodents (Supplementary Table S1). Spatial learning is a widely assessed task in preclinical research, mainly by using the Morris Water Maze [177], a model that allows the assessment of both short- and long-term spatial learning and memory. It is a task that has been extensively investigated at the cellular and molecular levels, and it has been fundamental for several important discoveries on learning, memory, neuronal plasticity, and related disorders [178,179,180]. A spatial learning improvement in rodents was also found by our search after treatment with extracts of *Punica granatum* and *Panax ginseng* (Supplementary Table S1). Human tests showed an effect on global cognition for the former and on memory for the latter (Table 1), findings that suggest an analogy with preclinical data [181].

Different findings were observed when comparing laboratory animal vs. human studies on *Dioscorea batatas* and *Melissa officinalis*. For both plants, preclinical studies showed a general effect on memory processing (Supplementary Table S1). On the other hand, human evidence on the two plants indicated changes in language performance and math processing, respectively (Table 1), functions that cannot be tested in laboratory animals.

The question that arises from our review is how to translate the findings of our cross-comparison between the laboratory data obtained in humans and the data obtained with preclinical neuropsychological tasks to the clinic. We think that before considering or proposing clinical trial protocols to demonstrate the pharmacological properties of the plants, or part(s) of them, in patients, it is important to define the profile of the potential therapeutic use. In fact, a limited number of phytotherapies are currently approved for some specific indications as a treatment *per se* and/or as a co-adjuvant of existing pharmacological and non-pharmacological interventions [182]. For instance, the only two botanical drugs that have been approved for marketing as prescription therapies by the FDA are Veregen^®^, an antiviral epigallocatechin gallate-based ointment, and Mytesi™, an oligomeric mixture of catechins used for the symptomatic relief of non-infectious diarrhea in HIV/AIDS patients [183].

As far as the integration with existing pharmacotherapies is concerned, a few reports in the literature investigated the effects of the association of G. biloba with choline esterase inhibitors, such as tacrine and donepezil [184,185]. Canevelli et al. (2014) extracted and analyzed data from a large study of AD patients who received G. biloba in conjunction with cholinesterase inhibitors [186]. They found that at the 1-year follow-up, but not at 6 mth, the plant adjunct to the medication significantly improved Mini Mental State Examination (MMSE) compared to medication-only, but not on Alzheimer’s Disease Assessment Scale-Cognitive (ADAS-Cog) subscale score and Activities of Daily Living (ADL) scale. This study not only supports a specific effect of G. biloba in AD but also suggests that further studies may better characterize (by confirming or excluding) the therapeutic potential of the plant on specific cognitive functions. A similar rigorous approach should always be used for assessing the safety of a plant treatment, whether administered *per se* or in association with a typical drug of choice [187,188].

In general, we think that a direct comparison between plant extracts and existing, established pharmacotherapies for cognitive disorders is not possible because of the inherently different rationale of use. In terms of mechanism of action, drugs act specifically on certain receptors or enzymes in the brain, producing well-defined effects, whereas plant extracts contain a wide range of bioactive compounds that can interact with multiple biological systems, often in more complex ways. In fact, drugs are designed based on hypotheses to treat specific disorders that may include improvements in mood, concentration, and memory. On the other hand, plant effects are often associated with more general effects, such as improved general well-being, reduced stress, and increased energy, with positive effects on cognition.

Human research on plants and/or plant extracts reviewed here suffers from some key issues related to study participants, small sample size, and temporal clinical endpoint assessments of limited relevance for the implications on chronic, declining cognitive deficits in elderly patients. Firstly, most of the human studies used samples composed of young [15,17,19] or elderly [16,18,20,21] healthy subjects. Only three papers have involved participants with neurodegenerative diseases (Multiple Sclerosis [23], Alzheimer’s disease [22]) and elderly patients with Mild Cognitive Impairment [24]. Although the results are encouraging, the generalizability of the findings to subjects with dementia remains however limited. Secondly, many of the included studies have extremely small sample sizes. However, it should be noted that in some cases, these were pilot studies [24]; in others, the sample size was justified by power analysis [19,21,22], or specific statistical analyses (e.g., nonparametric analyses [23]) were adopted to minimize the small sample problem. More studies are therefore needed in clinical samples of adult and older patients, with the evaluation of phytotherapy treatment effect in the long term.

Potential mechanisms of action have been identified for some plants and plant extracts. Although these mechanisms provide a rationale for the behavioral effects in laboratory animals and for the findings on cognitive performance shown by studies in humans, the identification of molecular mechanisms known to be affected in the cognitive neurodegenerative pathology, as well as in the effects of nootropic drugs, do not necessarily help to clarify the therapeutic mechanism of action. In fact, the effects of nootropics (whether drugs or phytotherapy) in reversing, preventing, or merely slowing cognitive decline is a complex issue that includes factors such as causes, extent, and stage of the cognitive decline condition and the individual response by patients. This issue further remarks on the limitations of the data reviewed here, particularly in terms of the features of the human study protocol design and of the translatability of animal data.

A further limitation observed in most of the preclinical phytotherapy literature reviewed here is the absence of a dose-response study to establish a possible relationship between the different extract doses and the improvement of cognitive performance. Dose-response curve experiments could help to further characterize the plant of interest, indicating a range of safety and efficacy of the extract, as well as a comparison criterion with the pharmacologic profiles of other drugs.

Moreover, additional limitations are the lack of experiments with an extended timeline to determine the long-term efficacy of the plant extracts, as well as a prevalent inconsistency in the terminology associated with the neurocognitive models analyzed, such as D-galactose (or scopolamine)-induced dementia vs. amnesia or even aging. This inconsistency can also be noticed in the treatment features. Indeed, even if the use of diverse approaches among the studies could be informative for the development of human experiments and future clinical trial protocols, as mentioned above, the methodological differences among the studies, such as the rodent species, the kind of extract, the administration timeline or the route of administration used, could limit the clear interpretation of the efficacy results on cognition. Finally, in a few cases, information on the origin of the plant extract, the route of administration, or the sex of the rodent species used was missing.

Both efficacy and safety of the current phytotherapy derive from the traditional plant use experiences accumulated over the centuries. However, in current times, the safe, efficient, and reproducible use of plant extracts in phytotherapy should require the standardization of preparation methods from the original plant matrix and the titration of their bioactive components. Thus, despite not being mandated by current regulations, evidence-based phytotherapy should strive for a comprehensive understanding of all the components of herbal preparation and how these interact to contribute to in-vivo bioactivity. While quantitative techniques, such as HPLC-UV/vis, are suitable and widely used for titration, a thorough elucidation of a plant extract can only be achieved through untargeted metabolomics analysis. Metabolomics techniques with high resolution and sensitivity (e.g., UPLC-HR-MS) enable, in fact, deep chemical characterization of the plant extract [189] and allow the identification of minor components that might affect the bioactivity and bioavailability of the most abundant ones. Furthermore, coupling untargeted metabolomics with multivariate statistical analysis represents a powerful biochemometric approach to identifying metabolite markers characterizing different sample groups. For instance, in phytotherapy research, this approach allows the monitoring of the fate of a phytocomplex administered in vivo by tracing its constituents and/or their catabolites in different biological fluids and organs [190,191]. Once clarified which metabolites are bioavailable and able to reach target organs, these phytochemicals can be mixed in various combinations to create simplified phytocomplexes for bioactivity re-testing, thus enabling the study of potential synergies or antagonisms [192]. This is crucial for uncovering the mechanism of action of the herbal product, which, due to the complexity of the plant matrix, often points to multiple targets in the organism. The current challenges and the strategies to study synergies or antagonisms between phytochemicals supported by biochemometric techniques have been recently and exhaustively revised [9]. These approaches overcome the limitations of traditional bioactivity-guided fractionation, which proposes single compounds or a restricted number of compounds as bioactive candidates without considering the potential synergy of the phytocomplex.

Among the plants discussed in this review, the most extensively investigated at the clinical level for various physiopathologies is *Ginkgo biloba*. Despite the detailed chemical characterization of its phytocomplex, the molecular mechanisms underlying the observed beneficial effects remain elusive [193]. In addition to evidence highlighting the pharmacological effects of individual constituents within the phytocomplex [37], recent efforts have focused on investigating in vitro synergies among the phytochemicals responsible for their anti-inflammatory and antioxidant properties. Compounds such as ginkgolides, proanthocyanidins, and organic acids demonstrate synergistic effects when combined with ginkgo flavonoids, effectively inhibiting inflammation-related pathways [194,195]. Additionally, another study reported that the combination of ginkgolide B and protocatechuic acid reduced neuronal damage in a rodent model of Parkinson’s disease [196]. The use of network pharmacology approaches [197] may aid in formulating active combinations of phytochemicals; however, as pointed out by Caesar & Cech, untargeted approaches to identify molecular targets of synergy and unravel the mechanisms of synergistic (or antagonistic) actions are still in their early stages. Further research in this area is critically important.

In conclusion, considering that we can now experimentally assess the potential beneficial features for specific patient segments, we have the opportunity to improve the clinical efficiency of phytotherapy with stronger evidence supporting appropriate indications with a better-known benefit/risk ratio. To propose controlled clinical trials should not be perceived as a further regulatory restriction of phytotherapy use but rather as the opportunity for the development of rationale supporting a more robust approach to the treatment of cognitive disorders. Such an approach may lead to the inclusion of phytotherapy in the existing ‘therapeutic package’ of interventions for those disorders—such as AD, PD, and senile dementia—that severely affect the aging population. On the methodological side, further clinical studies should be designed by taking into consideration the already known properties of the specific plant in order to design experimental trials aiming at clinical objectives suggesting an improved therapeutic benefit. Although traditional clinical studies for drug approval are designed according to the randomized controlled phase I, II, and III criteria, other experimental approaches (e.g., observational and Proof-Of-Concept studies) may also further support the preclinical laboratory data for specific disorder use—whether as a single or drug-associated treatment.

## Figures and Tables

**Table 1:** Studies included in the final synthesis for review.

Author, Year	Plant and Dosage	Part Used and Preparation	Cognitive Function	Neuropsychological Test	Study Design and Method	Main Results
Tohda et al., 2017 [1]	*Dioscorea batatas* (50 mg/day) for 12 weeks, followed by 6 weeks washing out, followed by other 12 weeks of treatment	Tuber (yam); The preparation process is not explained (Dioscorea capsules prepared by Shiratori Pharmaceutical, Tokyo, Japan)	LTM, WM, visual-spatial abilities, language, attention	RBANS: List Learning, Story Memory; Figure Copy, Line Orientation, Picture Naming, Semantic Fluency, Digit Span, Digit Symbol Coding, List Recall, List Recognition, Story Recall, Figure Recall; MMSE	Placebo-controlled, randomized, double-blind, crossover study. 28 healthy participants (17 were given yam extract for 12 weeks and, after 6 weeks washout period, placebo for 12 weeks. 11 were given placebo for 12 weeks and, after 6 weeks washout period, yam extract for 12 weeks) were tested before and after treatment	From before to after the 12-week treatment period the diosgenin-rich yam extract consumption led to a significant increase in the RBANS total score.). This increase was age-dependent (47–81 years group significantly increased compared to placebo). Regarding the cognitive subtests, diosgenin-rich yam extract was found to significantly improve semantic fluency
Elsabagh et al., 2005 [2]	*Ginkgo biloba* (120 mg) experiment 1: single dose; experiment 2: 6-week period daily administration	Leaves; The preparation process is not explained (Ginkgo tablets prepared by Lichtwer Pharma, Marlow, UK)	Sustained attention, MLT, WM, executive functions	CANTAB: Word Presentation, Picture Presentation, Intra Dimensional/Extra Dimensional, Stockings of Cambridge, Spatial Working Memory, Pattern Recognition Memory, Spatial Recognition Memory, Word Recall, Picture Recall, PASAT	Placebo-controlled double-blind design. Experiment 1: students (26 exp. group, 26 control group with placebo) were tested 4 h later. Experiment 2: students (20 exp. group, 20 control group with placebo) were tested at baseline and after 6 weeks	Experiment 1: The acute dose of Ginkgo significantly improved performance on PASAT and Pattern-Recognition Memory Task. Experiment 2: No effect of the treatment
Mix & Crews, 2000 [3]	*Ginkgo biloba* (180 mg/d) for 6 weeks	Leaves; The preparation process is not explained (*G. biloba* extract EGb 761 manifactured by Schwabe, Karlsruhe, Germany Pharmaceuticals, Gmbh, Karlsruhe, Germany)	Verbal and non-verbal LTM and WM, attention, visual discrimination, executive functions	MMSE, Stroop Color and Word Test; Trail Making Test (Parts A and B); WMS-R: Logical Memory I and II, Visual Reproduction I and II	Double-blind, fixed-dose, placebo-controlled, parallel-group experimental design. Elderly healthy participants (24 exp. group, 24 control group with placebo) were tested before and after 6 weeks	Exp. group exhibited significantly more improvement on Stroop Color and Word Test by the end of treatment as compared control group
Santos et al., 2003 [4]	*Ginkgo biloba* (80 mg/day) for 8 months	Leaves; The preparation process is not explained (Ginkgo extract was prepared by Magister Medicamentos Ltd.a, Sao Paulo, Brazil)	Verbal and non-verbal LTM and WM, language, logical reasoning, mathematical processing, visual-spatial abilities, attention, executive functions	WAIS-R: Information, Digit Span forward and backward, Vocabulary, Arithmetic, Comprehension, Similarities, Picture Arrangement, Picture Completion, Block Design, Object Assembly, Digit Symbol; WMS-R: Information, Orientation, Mental Control, Logical Memory, Verbal Paired Associates; Corsi Block-Tapping Test; Rey-Osterrieth Complex Figure Test; WCST; Toulouse Pieron Concentrated Attention; Verbal Free Recall	Double-blind study with placebo. Elderly healthy male participants (23 exp. group, 25 control group with placebo) were tested before and after 8 months	The exp. group compared to control group significantly improved performance on Corsi Block-Tapping Test, Toulouse Pieron Concentrated Attention, Verbal Free Recall (not in number of words but in errors); WAIS-R (Vocabulary, Arithmetic, Comprehension, Similarities, Block Design, Object Assembly, Digit Symbol); WMS-R (Mental Control, Verbal Paired Associates); Rey-Osterrieth Complex Figure Test (only in delayed retrieval); WCST (only mean number of non-perseverative errors)
Kennedy et al., 2004 [5]	*Melissa officinalis* (300 mg, 600 mg), 2 separate single doses	Leaves; dried leaves extracted up to exhaustion in a 30:70 methanol–water mixture. The liquid extract is evaporated and homogenized. Dried glucose syrup and colloidal anhydrous silicon dioxide (to 7% and 3% of the final dried weight, respectively) are added	Mathematical processing, visual discrimination, auditory processing, memory	DISS battery: Mathematical Processing, Visual Monitoring, Auditory Monitoring, and Memory Search tasks	Double-blind, placebo-controlled, randomized, balanced crossover experiment. 18 healthy volunteers received two separate single doses (300 mg, 600 mg) and a placebo, separated by a 7-day washout period. The battery was administered after each dose	Increase in the speed of Mathematical Processing task (no accuracy reduction) in 300-mg dose vs. placebo
Sayeed et al., 2013 [6]	*Nigella sativa* (500 mg) twice daily for 9 weeks	Seeds; seeds were crushed with mortar and pestle (60 min), then they were passed through a stainless steel screen (mesh size #30; 20 min) and filled into empty hard gelatin capsule shells (size #0) using a manual capsule filling machine (20 min)	LTM, verbal WM, visual-spatial abilities, visual discrimination, selective attention, information processing speed, executive functions	WMS-R: Logical Memory Test; Wais-R: Digit Span Test; Rey-Osterrieth Complex Figure Test; Letter Cancellation Test, Trail Making Test; Stroop Color and Word Test	Double-blind, placebo-controlled, randomized experiment. Elderly healthy volunteers (20 exp. group, 20 control group with placebo) were tested before and after 9 weeks	Differences in exp. group vs. control over time in the score of Logical Memory Test-I and II, total score of Digit Span, 30 min delayed-recall, percent score in Rey-Osterrieth Complex Figure Test, time taken to complete Letter Cancellation Test, time taken in Trail Making Test-A and test-B, Stroop Test (except Part W-Time)
Mazza et al., 2018 [7]	*Olea europaea* (extravirgin olive oil 20–30 g/day) for 1 year	Fruits; The preparation process is not explained (oil prepared by Opipari and Torchia Companies, Calabria, Italy)	Orientation, attention, mathematical processing, language, memory	MMSE; ADAS: ADAS-Cog subscale	Randomized study. Elderly healthy participants (55 exp. group with Mediterranean Diet + extravirgin olive oil, 55 control group with Mediterranean Diet alone) were tested at baseline and after 1 year	Exp. group compared to control group showed higher reduction of ADAS-cog scores (improved test) after 1 year (adjusted for food groups which were diferent between groups)
Lee et al., 2008 [8]	*Panax ginseng* (4.5 g/d, 9 g/d) for 12 weeks	Roots; The preparation process is not explained (powdered and encapsulated by Nonghyup Co., Seoul, Republic of Korea)	Memory, visual-spatial abilities, language, and orientation	MMSE; ADAS	Prospective open-label study. Alzheimer patients (58 exp. group, 39 control group, 9 higher dose group) were measured at baseline, after 4 weeks and after 12 weeks	Exp. group improved MMSE (after 12 weeks) and ADAS-cog subscale (after 4 and after 12 weeks) compared to control group. No differences between exp. group and higher dose group at 12 weeks
Petrou et al., 2021 [9]	*Punica granatum* (125 mg/day)	Seeds; The preparation process is not explained (a nano-formulation of pomegranate seed oil, produced by Granalix Bio Technologies Ltd., Jerusalem, Israel, was used)	Information processing speed, mathematical processing, verbal and non-verbal memory	PASAT; BICAMS: Symbol Digit Modalities Test, CVLT-II, Brief Visuospatial Memory Test	Randomized, double-blind, crossover clinical trial. 30 patients with multiple sclerosis (15 -group A- were given Pomenagrade supplemention for the first three months and then placebo pills for 3 months. Group B the opposite. Then the two groups received Pomenagrade supplemention for 6 additional months) were tested at baseline, after 3 months, after 6 months	CVLT-II significantly increased at 3-months in the group of patients who were treated with GranaGard, as compared to baseline
Lee et al., 2017 [10]	*Vitis vinifera* (72 g/day) for 6 months	Fruits; The preparation process is not explained (the dried-grape powder was provided by the California Table Grape Commission, Fresno, CA, USA)	LTM, WM, language, executive functions, information processing speed, visual-spatial abilities, attention	ADAS-Cog; MMSE; Hopkins Verbal Learning Test-Revised; Benton Visual Retention Test; Rey-Osterreith Complex Figure Test copy and delayed; Boston Naming Test; Letter Fluency; Category Fluency (animal naming); Stroop Color and Word Test; Trail Making Test (Part A and B); WCST; WAIS-III: Digital Symbol, Symbol Speed, Block Design, Symbol Search Total, Letter-Number Sequencing, Digital Span; WTAR	Prospective, placebo-controlled, double-blind randomized trial design. Subjects with mild decline in cognition (5 exp. group, 5 control group with placebo) were tested at baseline and after 6 months	No significant differences in scores on the neuropsychological battery of tests between the two groups

Abbreviations: DISS: Defined Intensity Stressor Simulation; ADAS: Alzheimer Disease Assessment Scale; MMSE: Mini Mental State Examination; LTM: long-term memory; Exp. Group: experimental group; WM: working memory; WMS: Wechsler Memory Scale; WAIS-R: Wechsler Intelligent Adult Scale Revised; CANTAB: Cambridge Neuropsychological Test Automated Battery; PASAT: Paced Auditory Serial Addition Task; WCST: Wisconsin Card Sorting Test; RBANS: Repeatable Battery for the Assessment of Neuropsychological Status; WTAR: Wechsler Test of Adult Reading; BICAMS: Brief International Cognitive Assessment for Multiple Sclerosis; CVLT-II: California Verbal Learning Test II.

## Data Availability

No new data were created or analyzed in this study. Data sharing is not applicable to this article.

## Supplementary Materials

The following supporting information can be downloaded at: https://www.mdpi.com/article/10.3390/nu16183156/s1, Supplementary Material S1_clinical tests.docx; Supplementary Material S2_preclinical tasks.docx; Supplementary Table S1_preclinical evidence.docx.

## Award Number

Project code CN_00000033, Concession Decree No. 1034 of 17 June 2022 adopted by the Italian Ministry of University and Research, CUP B33C22000660001, Project title “National Biodiversity Future Center—NBFC”.
