# Supplementary material for: Whole Plant Extracts for Neurocognitive Disorders: A Narrative Review of Neuropsychological and Preclinical Studies
Source: Nutrients. 2024 Sep 18;16(18):3156. doi: 10.3390/nu16183156 (PMC11434991; doi:10.3390/nu16183156)
Supplement: Supplementary file 1 [file nutrients-16-03156-s001.zip › Supplementary Material S1_clinical tests.pdf]

Supplementary Material S1

Batteries for global assessment of cognitive functioning

**Alzheimer's Disease Assessment Scale (ADAS), cognitive subscale (ADAS-Cog)**		
**Subtest**	**Description**	**Cognitive Function**
Word Recall Task	The participant must recall a list of words previously read aloud.	Delayed verbal memory
Naming Objects and Fingers	The participant must name real objects presented in random order and then fingers.	Language
Following Commands	The participant must execute commands of increasing difficulty.	Language; praxis
Constructional Praxis	The participant must raw a figure from a model.	Praxis; visuospatial abilities
Ideational Praxis	The participant is given a letter, an envelope, a stamp and a pen, and must perform the steps to mail a letter.	Praxis
Orientation	The participant must correctly identify place and day of the week, date, month, season, year, time of the day.	Memory (orientation)
Word Recognition Task	The participant reads words aloud, which are then randomly mixed with words that it has not read. The participant must indicate, for each word, whether it had been previously shown or not	Verbal memory
Remembering Test Directions	The participant is asked if the words named are the previously read words.	Verbal memory
Spoken Language	Assessment of the clarity with which the participant speaks throughout the examination.	Language
Comprehension	Assessment of participant's ability to understand spoken language.	Language
Word-Finding Difficult	Assessment of participant's difficulty in finding the desired word in spontaneous speech.	Language
**Brief International Cognitive Assessment for Multiple Sclerosis (BICAMS)**		
Symbol Digit Modalities Test (SDMT)	The subtest presents a series of nine symbols, each paired with a single digit in a key at the top of a standard sheet of paper. Participant is asked to voice the digit associated with each symbol as rapidly as possible for 90 sec.	Sustained attention
California Verbal Learning Test (CVLT2)	The subtest begins with the examiner reading a list of 16 words. The participant listens to the list and reports as many of the items as possible. Then the list is read again followed by a second attempt at recall. Altogether, there are five learning trials. After 25 min there is a delayed recall trial as well as a yes/no recognition memory task.	Learning, language and verbal memory
Brief Visuospatial Memory Test (BVMTR)	Six abstract designs are presented for 10 sec., and then the participant renders the stimuli via pencil on paper manual responses	Visual/spatial memory
**Cambridge Neuropsychological Test Automated Battery (CANTAB), modules of: attention; executive function; memory**		
Match to Sample Visual Search (MTS)	The participant is shown a complex visual pattern. After a brief delay, a varying number of similar patterns are shown. Only one of these patterns matches the pattern in the centre of the screen, and the participant must indicate which one.	Attention, psychomotor speed
Rapid Visual Information Processing (RVP)	Digits 2 through 9 appear in pseudo-random order. Participant is asked to identify target digit sequences. When the participant sees the target sequence, it must respond by selecting the button in the centre of the screen as quickly as possible.	Sustained attention

Cambridge Gambling Task (CGT)	The participant is presented with a row of ten boxes: some are red, and some are blue. The ratio of red and blue boxes varies between stages but there is always one box that contains a yellow token. Participant must choose the box colour in which it thinks the token is hidden. In the assessed stages, participant start with 100 points and select a proportion of these points to bet on its decision. The current bet value, which is either incrementally increase or decrease (depending on the task variant selected) is shown. Participant presses a button when it shows the proportion of its score it would like to bet. These points are either added or taken away to total score, depending on its decision and where the token is actually hidden.	Executive functions
CognitionKit Digit Symbol Substitution (DSST)	The task presents to the participant an array of abstract symbols associated with numbers. When a particular number is highlighted, the participant must draw the corresponding abstract shape using its index finger on the smartphone. This is a timed subtest, so the participant must try to complete as many symbol-to-number matches within the allotted timeframe.	Executive functions
Intra-Extra Dimensional Set Shift (IED)	Two artificial dimensions are used in the subtest: pink shapes and white lines. In this task, participant must use feedback to work out a rule that determines which stimulus is correct. After six correct responses, the stimuli and/or rule changes.	Executive functions
Multitasking Test (MTT)	The subtest displays an arrow which can appear on either side of the screen (right or left) and can point in either direction. Each trial displays a cue at the top of the screen that indicates to the participant whether it must select the right or left button according to the “side on which the arrow appeared” or the “direction in which the arrow was pointing”. In some sections this rule is consistent across trials (single task) while in others it may change from trial to trial in a randomized order (multitasking). Some trials display congruent stimuli whereas other trials display incongruent stimuli.	Executive functions
One Touch Stockings of Cambridge (OTS)	The participant is shown two displays containing three coloured balls. There is a row of numbered boxes along the bottom of the screen. It must move the balls in the lower display to copy the pattern in the upper display. Three problems are presented: one each requiring two moves, three moves and four moves. Then, the participant is shown further problems and must work-out how many moves the solutions require.	Executive functions; working memory
Spatial Working Memory (SWM)	The subtest begins with several coloured squares (boxes) shown on the screen. The aim of this subtest is that by selecting the boxes and using a process of elimination, the participant should find one yellow token in each of several boxes, and use them to fill up an empty column on the right-hand side of the screen. The colour and position of the boxes used are changed from trial to trial to discourage the use of stereotyped search strategies.	Executive functions; Spatial Working Memory
Stockings of Cambridge (SOC)	The participant is shown two displays containing three coloured balls. There is a row of numbered boxes along the bottom of the screen. It must move the balls in the lower display to copy the pattern in the upper display. The balls are moved one at a time. The participant is instructed to make as few moves as possible to match the two patterns. Movement time is discounted in a distinct phase of task, during which participants simply repeat the moves done by the computer. The moves shown by the computer mimic the moves the participant made when originally solving the problem.	Executive functions
Stop Signal Task (SST)	The participant must respond to an arrow stimulus, by selecting one of two options, depending on the direction in which the arrow points. If an audio tone is present, the participant must withhold making that response (inhibition). The subtest consists of two parts: in the first part, the participant is introduced to the subtest and told to select the left-hand button when it	Executive functions (response inhibition)

	sees a left-pointing arrow, and the right-hand button when it sees a right-pointing arrow. There is one block of 16 trials for the participant to practice this. In the second part, the participant is told to continue selecting the buttons when it sees the arrows but, if it hears an auditory signal (a beep), it should withhold its response and not select the button. The task uses a staircase design for the stop signal delay (SSD), allowing the task to adapt to the performance of the participant, narrowing in on the 50% success rate for inhibition.	
Delayed Matching to Sample (DMS)	The participant is shown a complex visual pattern followed by four similar patterns, after a brief delay. The participant must select the pattern which exactly matches the sample. In some trials the sample and the choice patterns are shown simultaneously, in others there is a delay (0, 4 or 12 sec.) before the four choices appear.	Short-term visual memory
Digit Span (DGS): Forward Digit Span and Reverse Digit Span	The participant hears a sequence of digits and then immediately be prompted to verbally repeat the sequence, either as it was heard (forwards), or in reverse order (backwards). If the participant correctly responds, the next trial presents a longer sequence. The task terminates when participant incorrectly responded on three occasions at a span length.	Verbal working memory; attention; executive functions
Cognition Kit N-Back (NBX)	The participants view a stream of abstract shapes one after another. In the 1-Back variant, participant must determine if the stimulus currently on screen, matches the stimulus shown directly before. If there is a match, it should touch the screen; if there is not a match, it should make no response. The 2-Back version is similar, but the participant must compare the current stimulus on screen with its memory of the two previous stimulus.	Attention; working memory
Paired Associates Learning (PAL)	Boxes are displayed on the screen and are "opened" in a randomized order. One or more of them contains a pattern. The patterns are then displayed in the middle of the screen, one at a time and the participant must select the box in which the pattern was originally located. If the participant makes an error, the boxes are opened in sequence again to remind the participant of the locations of the patterns.	Visual memory; learning
Pattern Recognition Memory (PRM)	The participant is presented with a series of visual patterns, one at a time, in the centre of the screen. In the recognition phase, the participant is required to choose between an already seen pattern and a novel pattern. In this phase, the subtest patterns are presented in the reverse order to the original order of presentation. This is then repeated, with new patterns. The second recognition phase is administered after a delay period, typically 10-20 minutes.	Immediate and delayed visual memory
Spatial Span (SSP)	White squares are shown on the screen, some of which briefly change colour in a variable sequence. The participant must then select the boxes which changed colour in the same order that were displayed by the computer (for the forward variant) or in the reverse order (for backward variant). The number of boxes in the sequence increases from two at the start of the subtest, to nine at the end, and the sequence and colour are varied through the subtest.	Visuospatial working memory
Verbal Paired Associates (VPA)	Participant is presented with a spoken set of eight pairs of words. It is then asked to remember which target word was paired with each prompt word. If the participant does not correctly recall all eight pairs, it is given up to two further attempts. There is a delayed recall variant of the task which may also be included to assess ability to recall the learned word pairs without hearing the list again.	Verbal memory
Verbal Recognition Memory (VRM)	The participant is shown a sequence of words on screen one by one. The participant is then tasked with recalling the words. In the next phase, the	Verbal memory; learning

	participant is presented with two words, one from the original list and one distractor, and it is asked to choose which one it has seen before, in a 2-force choice paradigm. The latter recognition phase is then repeated after a delay.	
**Defined Intensity Stressor Simulation (DISS), 4-modules computerized version**		
Mathematical processing	A series of calculations are presented. The participant adds two numbers, entering the 3-figure answer via an onscreen number pad. On completion of each calculation, the participant clicks on the "done" button, which cues the next calculation.	Battery tasks are administered simultaneously to induce cognitive-load stress. However, the individual tasks can be considered measures of mathematical processing; visual and auditory discrimination and memory
Visual monitoring	A small dot drifts outwards from the centre of a target comprising five concentric circles. The participant is instructed to allow the dot to travel as far out of the centre as possible, without letting it hit the edge of the target, before clicking on the "reset" button.	
Auditory monitoring	One of two tones of different pitches is sounded approximately every 5 seconds throughout the session in a random order. Participant is instructed to click on the box labelled "incoming mail" every time it hears the higher pitched of the two tones.	
Memory search tasks	Four letters appear to the participant to be remembered. After 4 seconds, the letters disappear but can be viewed again by clicking on "retrieve list" button. Approximately every 10 seconds, a single target letter appears. Participant is instructed to indicate whether the target letter had appeared in the original list of four letters by clicking on the "yes" or "no" buttons.	
**Mini-Mental State Examination (MMSE)**		
Immediate memory	Name 3 objects (one second for each). Then ask the participant to name all 3.	Immediate verbal memory
Temporal orientation	Correctly identify day of the week, date, month, season, and year.	Memory (orientation)
Spatial orientation	Correctly identify the country, the region, the city, the building, and the floor.	Memory (orientation)
Delayed recall	The participant must recall the 3 objects repeated above.	Delayed verbal memory
Attention and Calculation	Serial 7 task: participant counts down from one hundred by sevens. Alternatively, spell "world" backward.	Working memory
Naming	The participant must name the body parts indicated by the examiner.	Language
Verbal repetition	The participant must repeat a sentence.	Immediate verbal memory
Verbal comprehension	The participant must understand and execute a voice command consisting of three steps.	Working memory
Reading a sentence	The participant must read and carry out a simple instruction.	Working memory; language
Writing	The participant must write a sentence.	Language
Constructional praxis	The participant must copy a drawing.	Visuospatial and constructional abilities
**Repeatable Battery for the Assessment of Neuropsychological Status (RBANS)**		
List Learning	The examiner reads 10 semantically unrelated words aloud. The participant is asked to repeat as many words as possible after each of four learning trials.	Immediate verbal memory
Story Memory	A twelve-item short story is presented over two trials. After each presentation the participant is asked to recall as much of the story as it can remember.	Immediate verbal memory
Figure Copy	The subtest involves copying a geometric figure comprising ten parts, each evaluated for correctness and completeness on the one hand and proper	Visuospatial and constructional abilities

	location in relation to the rest of the figure on the other hand. The figure remains visually available while copying	
Line Orientation	A series of 13 identical lines, radiating out from a single point and spanning 180 degrees, are shown. Below this semi-circular, fan-shaped pattern of numbered lines (1–13), two lines of equal length that match two of the lines from the array are displayed. The participant is asked to give the numbers or point to the two lines that are identical in orientation to the two target lines.	Visuospatial and constructional abilities
Picture Naming	Ten line drawings are to be named by the participant. In case of an obvious misperception, a semantic cue is given.	Language
Semantic Fluency	The participant is asked to retrieve exemplars of a given semantic category within 60 sec.	Language
Digit Span	A string of digits is read aloud. The participant is asked to repeat the string in the same order. The length of the string increases by one, from two to nine digits. Two strings are provided for each length, but the second string of the same length is only read if the first one failed.	Attention; Verbal working memory
Coding	A page filled with symbols is presented to the participant and it is asked to fill in the number corresponding to each symbol using the key on top of the page. In this key the nine simple symbols are represented horizontally with the corresponding number (1– 9) underneath it.	Attention
List Recall	The participant is asked to recall as many words as it can remember from the list of 10 words learned previously in the List Learning subtest.	Delayed verbal memory
List Recognition	Twenty words are presented to the subject of which 10 words were on the original list (targets) and 10 were not (distractors). The participant is asked to declare whether each word was on the original list or not.	Delayed verbal memory
Story Recall	The participant is asked to recall as many details as it can from the story learned in the Story Memory subtest.	Delayed verbal memory
Figure Recall	The participant is asked to draw all the elements of the figure from the Figure Copy subtest that it can recall without visual display of the figure.	Delayed visual memory
**Wechsler Adult Intelligence Scale Revised (WAIS-R)**		
Information	The participant must answer general questions about information acquired from culture.	Language (verbal comprehension)
Comprehension	The participant must answer questions about social conventions, rules, and abstract expressions.	Language (verbal comprehension)
Arithmetic	The participant must solve mental mathematical problems.	Working memory; mathematical processing
Digit Span	A string of digits is read aloud. The participant is asked to repeat the string in the same order.	Attention; Verbal working memory
Similarities	The participant must identify the similarity between two things.	Language (verbal comprehension); logical reasoning
Vocabulary	The participant must answer questions focused on vocabulary comprehension.	Language (verbal comprehension)
Picture Arrangement	Presentation of cards in an incorrect order that the participant must place in the correct order to tell a story that makes sense.	Perceptual reasoning

Picture Completion	The participant must identify the missing part in a set of figures.	Perceptual reasoning; visual discrimination
Block Design	The participant must rearrange blocks that have various colour patterns on different sides to match a pattern.	Perceptual reasoning; visuospatial abilities
Object Assembly	The participant must arrange the presented pieces as in a puzzle.	Perceptual reasoning
Digit Symbol	Presentation of digit-symbol pairs followed by a list of digits whose corresponding symbol the participant must write down.	Processing speed; working memory; visuospatial abilities
**Wechsler Memory Scale Revised (WMS-R)**		
Mental Control	The subtest consists of three items: counting backwards from 20 to 1, counting by threes beginning with 1 up to 40, and reciting the alphabet.	Verbal working memory
Figural Memory	To the participant is shown a set of abstract designs, and after each set is removed, it is asked to identify the designs within a group of larger designs.	Visual memory, learning
Logical Memory I	The participant is told a brief story and then it is asked to retell the story.	Immediate verbal memory
Visual Paired Associates I	The participant is required to learn the colour associated with each of six abstract line drawings. Up to six learning trials were presented until the participant answers all six items correctly.	Visual memory
Verbal Paired Associates I	The subtest consists of eight paired associates (four are easy associates, four are hard associates). The participant is given up to six trials to learn the pairs to the criterion of one perfect repetition.	Immediate verbal memory
Visual Reproduction I	The subtest comprises four items/cards, three that contain a single geometric figure/design and a fourth card on which there are printed two designs. Each card is presented to the participant and after each presentation, it is requested to draw from memory each figure it recalls being on the card.	Immediate visual memory
Digit Span	The participant hears a sequence of digits and then immediately is prompted to verbally repeat the sequence, either as it was heard (forwards), or in reverse order (backwards).	Verbal working memory; attention
Visual Memory Span	The participant is asked to touch a series of coloured squares in a predetermined order, which was first demonstrated by the examiner.	Visual working memory
Logical Memory II	The participant is asked to repeat the story of the Logical memory I subtest after a 30-minute delay.	Delayed verbal memory
Visual Paired Associates II	This subtest consists of a delayed-recall trial 30 minutes after the administration of the Visual Paired Associates I subtest.	Delayed visual memory
Verbal Paired Associates II	The subtest consists of a delayed-recall trial 30 minutes after the administration of the Verbal Paired Associates I subtest.	Delayed verbal memory
Visual Reproduction II	The participant is requested to draw from memory all the designs that had initially been presented to it 30 minutes earlier in the Visual Reproduction I subtest.	Delayed visual memory

Table 2. Tests that measure specific cognitive functions

Test	Description	Cognitive function
Benton Visual Retention Test (BVRT)	The participant is shown ten designs, one at a time, and asked to reproduce each one as exactly as possible on plain paper from memory. These designs can be administered in several ways: changing the observation time (5 or 10 seconds); leaving or not leaving the pattern visible during copying; making the participants wait 15 seconds before copying the drawing; choosing from a range of patterns the one originally seen.	Visuospatial working memory; visuospatial and constructional abilities
Boston Naming Test (BNT)	The task consists of 60 drawings of objects of graded difficulty, ranging from very common objects to less familiar objects. The participant is shown target stimuli and asked to identify each target item within a 20-second interval per trial. If it fails to give the correct response initially, the examiner provides a phonemic cue, which is the initial sound of the target.	Language (word retrieval ability)
Category Fluency: Animal Naming	The participant must generate as many animal names as possible in 60 seconds.	Semantic memory; language
Corsi block-tapping test (CBT)	Nine blocks are affixed to a board. An examiner uses a stick to tap out a sequence of blocks while the participant observes. The participant then attempts to tap out the sequence they just observed. The sequence starts out simple, usually using two blocks, but becomes more complex until the participant performance fails.	Visuospatial working memory
Hopkins Verbal Learning Test-Revised (HVLT-R)	It is a list learning test, which consists of 12 nouns within three semantic groups. The acquisition variable consists of three acquisition trials in which the administrator reads the words aloud and then asks the participant to repeat as many as it can remember in any order. A delayed recall trial is introduced after 20–25 min, in which the participant is asked to retrieve as many of the words listed in the acquisition trial as it can remember.	Verbal learning; memory (acquisition and delayed recall)
Letter Cancellation Test (LCT)	Patterns of letters interspersed with a target letter are shown to the participant, with the instruction to cross out (cancel) all the targets while ignoring similar non-target distracter items. Task difficulty can be changed by varying the spacing and frequency of the targets.	Selective attention and visual discrimination
Letter Fluency	The participant must generate words cued with a specific letter within time limits (generally, 60 seconds).	Memory; language
Rey-Osterrieth complex figure test (ROCF)	The participant is asked to reproduce a complicated line drawing, first by copying it freehand (recognition), and then drawing from memory (recall).	Visuospatial abilities; working memory; executive functions.
Stroop Colour and Word test (SCWT)	Participant is required to read three different tables as fast as possible. Two of them represent the "congruous condition" in which participant is required to read names of colours printed in black ink and name different colour patches. In the third table, colour-words are printed in an inconsistent colour ink (for instance the word "red" is printed in green ink). Participant is required to name the colour of the ink instead of reading the word.	Executive functions
Toulouse Pieron Concentrated Attention (TP)	The test consists of a blank sheet of paper with twenty-five lines and forty small squares per line. The squares are distinguished from each other through the orientation of the rows on the outer surface: in each square the stroke is oriented in eight possible directions. The participant is required to cross out three targets presented in the header.	Selective attention; sustained attention
Trail making test-A and test-B (TMT)	In Part A, the participant must draw a line to connect consecutive numbers, from 1 to 25. In Part B, the participant connects numbers and letters in an alternating progressive sequence, 1 to A, A to 2, 2 to B, and so on.	Executive functions

Wisconsin Card Sorting Test (WCST)	The test consists of 4 stimulus cards and 128 response cards (2 decks of 64 cards), on which figures vary in: number (1 to 4 per card); shape (circles, triangles, crosses, or stars); colour (red, blue, yellow and green). Cards are shown to the participant and the participant is then instructed to match the cards. No instructions are given on how to match the cards, only feedback when the matches are right or wrong. The participant must discover the correct classification criterion. During the test, the criterion will be changed without warning, requiring a new classification strategy to be developed.	Executive functions
Wechsler Test of Adult Reading (WTAR)	The test is composed of 50 irregularly spelled words. The examiner begins by presenting the first word card and prompting the participant for a single pronunciation of the word. This procedure continues through all 50-word cards and is discontinued if the patient provides 12 consecutive incorrect pronunciations.	Intelligence (pre-morbid); memory (pre-morbid)
