# Supplementary material for: Whole Plant Extracts for Neurocognitive Disorders: A Narrative Review of Neuropsychological and Preclinical Studies
Source: Nutrients. 2024 Sep 18;16(18):3156. doi: 10.3390/nu16183156 (PMC11434991; doi:10.3390/nu16183156)
Supplement: Supplementary file 1 [file nutrients-16-03156-s001.zip › Supplementary Material S2_preclinical tasks.pdf]

Supplementary Materials S2: Preclinical tasks

- 8-arm radial maze (8-ARM)

The 8-ARM apparatus consists of an octagonally shaped centre and 8 radially extended closed arms. For training, half of the arms are reinforced and animals can visit the arms in a given time. At test, the same arms are reinforced, and entering into a non-reinforced arm was indexed as a reference memory error, while re-entering an arm in which the reinforcement was already taken was indexed as a working memory error.

- Y-maze (and T-maze)

The Y-maze apparatus consists of 3 identical arms, and the animal is allowed to move freely in the maze for a given time. Spontaneous alternation percentage, defined as the entries into each of the three different arms consecutively, is considered an index of working memory in animals. As Y-maze, also T-maze has 3 identical arms; however, the more gradually angled arms in Y-maze allow for a faster layout comprehension of the maze.

- Barnes maze task (BMT)

The BMT apparatus is a white large circular platform with a number of holes at its edge, and visual signs as spatial cues. One hole leads to an “escape box”, allowing animals to hide from the light. For training, animals left in the platform centre have to find the escape hole during multiple sessions. At test, the position of the escape box is changed, and the latency to find the escape box, as well as the n° of wrong holes explored are assayed as spatial learning and memory correlates.

- Elevated plus maze (EPM)

Here, EPM was used for spatial memory evaluation. The apparatus, elevated from the floor, consists of a "+"-shaped maze with 4 arms, 2 open and 2 closed, and a central zone. Acquisition and retention of spatial learning and memory was evaluated using the transfer latency (TL) parameter, i.e., the time needed by the animal to move from the open to the closed arm(s). A decrease of TL between training and test sessions is indicative of spatial memory retention.

- Morris water maze (MWM)

Used to investigated long-term spatial learning and memory, MWM consists of a large circular pool filled with opaque water. Visual signs could be placed as spatial cues. An escape platform is placed in a given position and hidden under the water. For training, the animal is placed in the pool and tasked to find the platform in a given time, along multiple sessions. The decrease in latency to find the platform indicate spatial learning. For testing, the animal is free to swim in the platform-free pool, and the latency to and the time spent in the previous platform location indicate long-term spatial memory.

- Novel object recognition task (NORT)

NORT is performed in an open arena. For habituation, animals are free to explore the arena. During training, animals can explore 2 identical objects in the arena for a given time. At test, a novel object replaces one of the explored “familiar” object and recognition memory is evaluated by measuring the percentage of time spent exploring the novel vs. the familiar object.

- Olfactory recognition task

Similar to NORT, 2 different odours are used instead of 2 objects. For habituation, animals are free to explore the arena. At training, one odour (O1) is presented in the arena and animals are free to sniff the odour for a given time. At test, a second odour (O2) is added, and the time spent sniffing O2 vs O1 is a measure of recognition memory. Short or long delay between training and test are used to evaluate short- or long-term memory.

- Fear conditioning

To test for associative fear learning and memory. For cued fear memory test, animals associate a visual (or auditory) cue to a mild electrical footshock after multiple paired presentation. Associative learning and memory is tested placing the animal in a new chamber with the same cue, and the freezing time is measured. For contextual fear memory, the association is between the chamber and the footshock, with no cues, and freezing time is tested during chamber exposure.

- Active (AA) and passive (PA) avoidance tasks

These are fear-motivated associative learning tasks. Here, the animals are trained to associate a conditioning chamber to a negative consequence, like a footshock. For AA, animals can avoid the footshock by moving from the conditioning chamber to a different compartment in a given time. For PA, animals are placed in an unpleasant situation, like a heavily lit chamber close to the conditioning one, and can avoid the footshock by remaining in the unpleasant situation.

- Conditional visual discrimination task

A test to measure the visual learning and cognitive flexibility of animals in operant boxes, with position signal discrimination and colour visual signal discrimination sub-tasks. In the first sub-task the animals learn to discriminate between a reinforced cued lever and a non-reinforced level, regardless of cue (light). In the second sub-task, the cues were 2 different lights, one signalling a reward in the previous reinforced lever, and one associated to no reward regardless the lever pressed.

- Operant discrimination task

The operant discrimination task is performed in an operant box equipped with 2 levers, and animals learn to press the lever corresponding to a turned on-cue light. Pressing the lever corresponding to the cue light is reinforced. This test measures associative learning and memory. To test for behavioural adaptation, a stressor is added during training and/or test.
