# Supplementary material for: Whole Plant Extracts for Neurocognitive Disorders: A Narrative Review of Neuropsychological and Preclinical Studies
Source: Nutrients. 2024 Sep 18;16(18):3156. doi: 10.3390/nu16183156 (PMC11434991; doi:10.3390/nu16183156)
Supplement: Supplementary file 1 [file nutrients-16-03156-s001.zip › Supplementary Table S1_preclinical evidence REV.pdf]

Table S1: Summary of the behavioural effects induced by *Dioscorea batatas*, *Ginkgo biloba*, *Melissa officinalis*, *Nigella sativa*, *Olea europaea*, *Panax ginseng*, *Punica granatum*, and *Vitis vinifera* extracts on the different cognitive decline models accounted in the review. The table follow the order of the articles discussed in the review, indexed by the investigated plants and then by the animal models used by the Authors.

Author, Year	Species	Model	Plant and Dosage	Test	Main results
[15] Tohda et al., 2017	Male and female ddY mice	Naïve or 5xFAD tg male and female mice	*Dioscorea batatas*<br>Diosgenin or Diopower 15 (purchased).<br>1: diosgenin 4.14 mg/kg/d for 5d<br>2: diosgenin 0.0414 mg/kg/d for 4d<br>3: Diopower 15 0.259 mg/kg/d for 7d<br>4: diosgenin 0.0414 mg/kg/d for 20d	NORT	Diosgenin, dissolved in a water-based solution, 4.14 mg/kg/d enhanced NOR memory in normal ddY male mice.<br>Diosgenin 0.0414 and Diopower 15 0.259 mg/kg, dissolved in an oil-based solution, enhanced NORT memory normal ddY male and female mice.<br>Diosgenin, dissolved in an oil-based solution, 0.0414 mg/kg/d for 20d p.o. enhanced object recognition memory in 5xFAD male and female mice.
[31] Tian et al., 2012	Male and female SD rats	AD model: Aβ25–35 1μg/μL, 1 μL/min in the rat hipp for 5 min	*Ginkgo biloba*<br>EGb761 (purchased)<br>20 mg/kg/d, p.o. for 20d	MWM	EGb761 reduced the escape latency, and increased the entries in and the swimming time in the target quadrant in the MWM, compared to AD model-vehicle treated rats.
[32] Tian et al., 2013	Male SD rats	AD model: Aβ25–35 1μg/μL, 1 μL/min in the rat hipp for 10 min	*Ginkgo biloba*<br>EGb761 (purchased)<br>20 mg/kg/d p.o. for 20d	MWM	EGb761 reduced the escape latency, and increased the entries in and the swimming time in the target quadrant in the MWM, compared to AD model-vehicle treated rats
[33] Zhang et al., 2015	Male SD rats	AD model: Aβ25–35 1μg/μL, 1 μL/min in the rat hipp for 10 min	*Ginkgo biloba*<br>EGb761 (purchased)<br>20 mg/kg/d p.o. for 20d	MWM	EGb761 reduced the escape latency, and increased the entries in and the swimming time in the target quadrant in the MWM, compared to AD model-vehicle treated rats
[34] Tu et al., 2020	Male Wistar rats	AD model:<br>icv injection bilaterally of 10μg of the Aβ1-42	*Ginkgo biloba*<br>EGb761 (purchased)<br>20 mg/kg/d p.o. for 23d	MWM	EGb761 increased the swimming speed and the number of target platform crossings in the MWM, compared to AD model vehicle-treated rats
[35] Hoyer et al., 1999	1-year-old male Wistar rats	AD model:<br>bilateral icv of STZ 0.25mg/2mL, 3 times	*Ginkgo biloba*<br>EGb761 (made in lab)<br>50mg/d, with standard food, for 80d	PA	In the PA, EGb761 only partially restored the acquisition and retrieval of memory of aversive shock damaged by STZ, compared to vehicle-treated rats.
[36] Liu et al., 2015	Female mice with C57BL/6 background (bkg)	AD model: TgCRND8 APP-transgenic mice	*Ginkgo biloba*<br>EGb761 (kindly provided)<br>600 mg/kg with diet.<br>2-m-o mice treated for 5 months or 5-m-o mice treated for 2 months	BMT	2-month-old mice treated with EGb761 for 5 months, but not the 5-month-old mice treated for 2 months, spent less time and travelled a shorter distances to reach the escape vs. mice receiving the control diet.
[37] Qin et al., 2018	Male and female mice with C57BL/6 and C3H/HeJ background	AD model:<br>tau-transgenic mice overexpressing human tau mutant P301S	*Ginkgo biloba*<br>EGb761 (kindly provided)<br>600 mg/kg with diet<br>4-m-o mice treated for 5 months or 7-m-o mice treated for 2 months	MWM	4-month-old mice treated with EGb761 for 5 months, but not the 7-month-old mice treated for 2 months, spent less time and travelled a shorter distances to reach the escape vs. mice receiving the control diet.
[38] Liu et al., 2022	Male mice	AD model:<br>APP/PS1 transgenic mice (B6C3-Tg)	*Ginkgo biloba*<br>Leaf extract (GBLE, purchased)<br>40 mg/mL of EGb761 delivered at 50 mg/kg/d for 90d p.o.	MWM	GBLE reduced the escape latency, and increased the number of platform crossing, the distance travelled in the target quadrant and the time in the target quadrant compared to vehicle-treated APP/PS1 mice.
[39] Wan et al., 2016	Male mice	AD model:<br>APP/PS1 transgenic mice	*Ginkgo biloba*<br>EGb761 (company, purchased)<br>50 mg/kg/d for 6 months, with diet	MWM	EGb761 reduced the escape latency and the time required to pass the old platform location, and increased the number of platform crossing at test, compared to APP/Psi vehicle-treated mice.

[40]<br>Stackman et al., 2003	Female mice with C57BL/6 and SJL bkg	AD model:<br>Tg2576 transgenic mice	*Ginkgo biloba*<br>Extract similar to EGb761 (purchased)<br>70 mg/kg/d for 6 months, in water	MWM	*Gb* only reduced the number of trials to learn the water maze task compared to water-treated Tg2576 mice
[41]<br>Ge et al., 2021	Male mice	AD model:<br>5xFAD transgenic mice	*Ginkgo biloba*<br>EGb761 (purchased)<br>0, 10, 20, 30 mg/kg i.p. for 4 months	NORT<br>MWM	EGb761 30 mg/kg enhanced preference index for the new object in the NORT, compared to 5xFAD mice.<br>EGb761 20 and 30 mg/kg reduced the escape latency, and increased the number of platform crossing and the time spent in the target quadrant time in MWM, compared to 5xFAD mice.
[28]<br>Shao et al., 2021	Male mice	Aging: 6-m-o senescence-resistant R1 (SAMR1) and senescence-accelerated P8 (SAMP8) mice	*Ginkgo biloba*<br>Ginkgolide B (GB, purchased)<br>20, 30 or 40 mg/kg/d p.o. for 3 weeks	MWM<br>NORT	GB 20, 30, 40 mg/kg significantly shortened latency to platform during training and test, and increased swimming time in the target quadrant in the MWM, for SAMP8-treated vs. SAMR1 control mice.<br>GB 30 and 40 mg/kg significantly increase the discrimination index in the NORT, for SAMP8-treated vs. SAMR1 control mice.
[42]<br>Zeng et al., 2018	Male SD rats	AD model:<br>HHcy with Hcy (400 μg/kg/d) for 14d	*Ginkgo biloba*<br>EGb761 (purchased) 400mg/kg/d p.o.<br>1: prevention, with simultaneous Hcy and EGb761 for 14d<br>2: treatment, with EGb761 for 7d	MWM, Fear conditioning	EGb761 reverted the deficits in the escape latency in MWM, and in the short- (1h) and long-term (24h) freezing time in fear conditioning, both as preventive and treatment intervention compared to HHcy vehicle-treated rats.
[43]<br>Koz et al., 2012	Female Wistar rats	AD model:<br>HHcy with 1 g/kg methionine in water during pregnancy	*Ginkgo biloba*<br>EGb761 (purchased)<br>100mg/kg p.o. twice per day, throughout pregnancy	MWM	EGb761 during pregnancy did not improve performances (escape latency, swim distance and the time in the target quadrant) in MWM, compared to HHcy vehicle-treated rats.
[45]<br>Dong et al., 2023	Male C57BL/6 mice	VD model: MCAO	*Ginkgo biloba*<br>Novel Gb leaf extract (nGBE, purchased)<br>5.25, 10.5 or 21 mg/kg i.v. for 14d	MWM, NORT	nGBE decreased the escape latency time and increased the time in the target quadrant in the MWM, compared to MCAO vehicle-treated mice.<br>nGBE increased the time spent in exploring the novel object in the NORT, compared to MCAO vehicle-treated mice.
[46]<br>Kwak et al., 2012	Male Wistar rats	VD model: 2-VO	*Ginkgo biloba*<br>Gb extract (GB, purchased)<br>20 mg/kg/d p.o. for 21d after surgery	8-ARM	GB reduced the total error score, the time spent in the maze arms and the reference memory error score compared to the control group.
[47]<br>Vaghef and Gharamaleki, 2017	Male Wistar rats	VD model: 2-VO	*Ginkgo biloba*<br>Gb extract (purchased) similar to EGb761<br>100mg/kg/d for 2 weeks before 2-VO	MWM	GB reduced the escape latency, and increased the time spent in the target quadrant in the MWM after 2-VO, compared to 2-VO vehicle-treated rats.
[48]<br>Yao et al., 2021	Male SD rats	VD model: 2-VO	*Ginkgo biloba*<br>EGb761 (purchased)<br>100 mg/kg i.p. for 1 month after 2-VO	MWM<br>NORT	EGb761 after 2-VO decreased the escape latency, and increased the platform crossing time and the time spent in the target quadrant in MWM, both at training and test, compared to 2-VO model.<br>In NORT, EGb761 after 2-VO increased the time spent in exploring the novel object, compared to 2-VO model.
[49]<br>Harada et al., 2015	Male ddY mice	VD model: 2-VO	*Ginkgo biloba*<br>Gb leaf extract (GB, purchased)<br>100, 200, 400 mg/kg/d p.o. for 5d	PA	GB 100, 200 and 400 mg/kg dose-dependently decrease the response latency in the PA test after 2-VO, compared to control.
[50]<br>Wang et al., 2013	Male SD rats	VD model: sodium nitroprusside i.p. 2.5 mg/kg + 2-VO	*Ginkgo biloba*<br>EGb761 (purchased)<br>50 mg/kg p.o. acute	MWM	EGb761 reduced the escape latency at 15 days, and at 1, 2 and 4 months after 2-VO, compared to vehicle-treated 2-VO model.

[51] Paganelli et al., 2006	Male Wistar rats	VD model: 4-VO	*Ginkgo biloba* EGb761 (gifted) 50 or 150 mg/kg p.o. for 4d, 3 doses/day	Aversive 8-ARM	EGb761 150 mg/kg improved the 8-ARM performances, i.e. latency, reference memory errors, and working memory errors, during acquisition but not at retention tests.
[52] Domoráková et al., 2009	Male Wistar rats	VD model: 4-VO	*Ginkgo biloba* EGb761 (purchased) 40 mg/kg p.o. acute	MWM	EGb761 did not improve the latency time for reaching the escape platform at the MWM, when administered before or after 4-VO, compared to control.
[27] Huang et al., 2021	Male SD rats	VD model: 2-VO	*Ginkgo biloba* Ginkgolide B (GB, purchased) 1.0mL/d at 1.0mg/mL i.p. for 14d	MWM Y-maze (electric maze)	GB reduced the escape latency and increased the active avoidance response number compared to vehicle-treated VD rats, in the Y-maze. GB reduced the escape latency and improved spatial learning and memory decline compared to vehicle-treated VD rats, in the MWM.
[29] Li et al., 2013	Male SD rats	VD model: 2-VO	*Ginkgo biloba* Bilobalide (BB, purchased) 2, 4 or 8 mg/kg p.o. for 60d after VD	MWM	BB 2, 4, 8 mg/kg decreased escape latency, the swimming time in the target quadrant, and the number platform crossing in the MWM, compared to vehicle-treated VD rats.
[53] Rapin et al., 1994	Male Long-Evans rats	Aging: 4 vs. 20-month-old	*Ginkgo biloba* EGb761 (provided) 50 or 100 mg/kg p.o. for 21d	Operant discrimina-tion task	EGb761 50 and 100 mg/kg suppressed the stress-induced disruption of learning, and the effect was more pronounced in 4 than in 20-month-old rats. EGb761 100 mg/kg reduced stress-induced increase in reaction time in 4-m-o rats, while 50 mg/kg was effective on 20-m-o rats.
[54] Stoll et al., 1996	female NMRI mice	Aging: 3, 12, and 22-24-month-old	*Ginkgo biloba* EGb761 (no origins or RoA info) 100 mg/kg/d for 3 weeks	PA	EGb761 attenuates the age-related changes in the PA short-term memory test, with significant effects in aged animals.
[55] Winter, 1998	Male Fischer 344 rats	Aging: 20 and 26-month-old	*Ginkgo biloba* EGb761 (purchased) 50, 100, 200 mg/kg p.o. lifetime treated	8-ARM	EGb761 dose-dependently decreased total, retroactive, and proactive errors in the 8-ARM on 20-m-o rats, and 200 mg/kg ameliorated the performance also on 26-m-o rats
[56] Wirth et al., 2000	Male and female Long–Evans rats	Aging: Exp 1: young male rats Exp 2: young male rats Exp 3: aged female rats	*Ginkgo biloba* EGb761 (purchased) Exp 1: 30 or 60 mg/kg/d for 30 days i.p. Exp 2: 60 or 120 mg/kg i.p. acute Exp 3: 60 mg/kg i.p. acute	Olfactory recognitio n task	EGb761 60 mg/kg for 30 days, and acute 60 and 120 mg/kg, increased the recognition index in the olfactory test on young male rats, compared to control. EGb761 enhanced, rather non significantly, the recognition index in the olfactory test on aged female rats, compared to control.
[57] Wang et al., 2006	Male Wistar rats	Aging: 74- to 78-weeks-old	*Ginkgo biloba* EGb761 (purchased) 30 or 60 mg/kg for 30d, p.o., with diet	MWM	EGb761 60 mg/kg reduced the escape latency and increased the distance travelled and the time spent in the target quadrant vs. aged untreated rats. EGb761 30 and 60 mg/kg improved cognitive flexibility in the platform-switched MWM vs. aged untreated rats.
[58] Belviranlı and Okudan, 2015	female Wistar rats	Aging: 18-month-old	*Ginkgo biloba* EGb761 (purchased) 100 mg/kg/d p.o. for 30 days	EPM MWM	EGb761 did not reduced the total distance, the time spent in the centre, and number of entries in closed, centre and open arms at the EPM test, vs. control. EGb761 only increased the number of platform crossings in MWM vs. control.
[59] Ward et al., 2002	C57BL/6 male mice	Aging: Exp 1: 20-m-o mice Exp 2: 3- or 10-m-o mice	*Ginkgo biloba* EGb761 (purchased) 100 mg/kg/d p.o. for 82 days	MWM, EPM	Exp1: no effects of EGb761 on 20-m-o mice in MWM acquisition or retention, nor in EPM total time spent on the open arms. EGb761 increased the total time spent on the open arms when the EPM was administered after a stressful experience (anti-anxiety buffer effect)
[60] Pardon et al., 2004	B6D2F1 female mice	Aging:17–18, 23–24-m-o Stress: chronic ultramild stress	*Ginkgo biloba* EGb761 (purchased) 50 mg/kg/d for 7 months, in water	T-maze	EGb761 reduced the impact of stress on evaluation and hesitation score of T-maze, in senescent mice.
[61] Chopin and Briley, 1992	Male SD rats	SCO-induced amnesia: acute 1 mg/kg i.p.	*Ginkgo biloba* EGb761 (purchased)	PA	EGb761 150, 300, 400 and 500 mg/kg significantly attenuated the amnesic effects of SCO 1 mg/kg

			50, 100, 150, 200, 300, 400, 500 mg/kg i.p. acute		
[62] Das et al., 2002	Male Swiss mice	SCO-induced amnesia: 3 mg/kg i.p.	*Ginkgo biloba* Similar to EGb761 (Ginkocer, purchased) 15, 30, 60 mg/kg p.o. for 7d, after SCO	PA	GB 30 and 60 mg/kg increased the transfer latency time in PA vs. SCO-treated mice
[63] Zhang et al., 2019	ICR mice (sex not specified)	SCO-induced amnesia: 3mg/kg i.p.	*Ginkgo biloba* Ginkgo ketoester tablets (GT): 58.5 mg/kg for 15 days	MWM	GT, alone or in combination with donepezil 0.65 mg/kg, decreased the escape latency in MWM vs. model mice. GT alone increased the number of platform crossing, and the distance ratio in the target quadrant when in combination with donepezil, in MWM vs. model mice.
[64] Zhao et al., 2021	Male Wistar rats	SCO-induced amnesia: 1mg/kg i.p. for 14d	*Ginkgo biloba* EGb761 (Tebonin, purchased) 8.27 mg/kg p.o. for 15d, 3 times/day	MWM	EGb761 alone decreased only the escape latency, while in combination with 1 mg/kg donepezil decreased escape latency and increased the time spent in target quadrant and the number of platform crossing vs. SCO model.
[65] Zhang et al., 2022	Male SD rats	SCO-induced amnesia: 1 mg/kg i.p.	*Ginkgo biloba* Gb extract similar EGb761 (purchased) 400 mg/kg/day p.o. for 14 days	MWM Fear conditioning	Gb reduced the escape latency and increased the number of platform crossing in MWM vs. SCO model. Gb increased the percentage of the freezing time in contextual and cue fear conditioning tests vs. SCO model.
[66] Hong et al., 2022	ICR male mice	SCO-induced amnesia: acute 1.2 mg/kg i.p.	*Ginkgo biloba* Gb extract similar to EGb761 (purchased) 40 mg/kg p.o. for 7d	Y-maze NORT	Gb rescued the spontaneous alternation percentage in Y-maze and the exploration times of the novel objects in the NORT to normal levels, vs. SCO model.
[67] Wang et al., 2013	SD rats (sex not specified)	D-gal-induced dementia: 100 mg/kg i.p. for 8 weeks	*Ginkgo biloba* Gb extract similar to EGb761 (purchased) Gb 0.875, 1.75 or 3.5 mg/kg i.p. for 8 weeks with D-gal	Y-maze (electric maze)	After 6 weeks of co-administration with D-gal, Gb 1.75 and 3.5 mg/kg reverted to control levels the trial and memory indices of Y-maze vs. D-gal model.
[68] Li, Zhang and Chen, 2019	Wister male rats	D-gal induced dementia: 0.5% D-gal 10 ml/kg/d s.c. for 6 wk	*Ginkgo biloba* EGb761 (purchased) 8.75, 17.5, or 35 mg/kg p.o. for 6 wk	MWM	All the 3 doses of EGb761 reduced the escape latency and increased the time in the target quadrant and the number of platform crossing in the MWM, vs D-gal model.
[26] Liu et al., 2021	Male C57BL/6 mice	D-gal + $AlCl_3$ induced dementia: D-gal 120 mg/kg and $AlCl_3$ 200 mg/kg p.o. for 6 weeks	*Ginkgo biloba* Ginkgolide B (GB, purchased) 0.1% Ginkgolide B p.o. for 2 weeks before AD induction, and for 2 weeks after AD induction.	NORT	GB administration to AD model mice for 4 weeks ameliorated the discrimination index in the NORT test compared to AD vehicle-treated group
[69] Yu, et al., 2021	Male C57BL/6 mice	Parkinson's disease model: MPTP 30 mg/kg/d i.p. for 5d	*Ginkgo biloba* EGb761 (purchased) Gb dropping pills (GBDP, purchased) EGb761 or GBDP 50 mg/kg/d for 14d	MWM	GBDP increased the number of platform crossing in the MWM, vs. MPTP model. EGb761 non-significantly increased the number of platform crossing in the MWM, vs. MPTP model.
[70] Adebayo et al., 2022	Male Swiss mice	Parkinson's disease model: rotenone (ROT) 1.5 mg/kg i.p. 1/2d before or after Gb	*Ginkgo biloba* GB extract similar to EGb761 (purchased) Preventive: Gb 20 mg/kg/d p.o. for 21d and ROT for the last 10d of Gb. Curative: ROT for 21 days and Gb 20 mg/kg p.o. for the last 10d of ROT.	Y-maze	Preventive treatment with GB, but not the curative treatment, increased the percentage of alternation in the Y-maze, vs. PD model
[93] Ozarowski et al., 2016	Male Wistar rats	SCO-induced amnesia: 0.5 mg/kg i.p. acute	*Melissa officinalis* MO leaves (purchased) Hydroethanolic extract	PA NORT	MO did not recover the decrease of short-term memory in the NORT induced by SCO scopolamine decreased short-term memory

			200 mg/kg p.o. for 28 days		Mo alone increased the long-term memory in the PA vs. control, but did not recover the SCO effects
[97] Abulfadl et al., 2018	Male SD rats	D-gal + $AlCl_3$ induced dementia: D-gal 60mg/kg/d i.p. + $AlCl_3$ 10mg/kg/d i.p. for 42d	*Nigella sativa*<br>Thymoquinone (TQ, purchased)<br>10, 20, and 40 mg/kg/d p.o. during the last 14d of D-gal/$AlCl_3$	PA	TQ 10, 20 and 40 mg/kg increased the latency to enter the dark compartment after 24 and 48h in PA vs AD model control rats, in PA.
[98] Abulfadl et al., 2018	Male SD rats	D-gal + $AlCl_3$ induced dementia: D-gal 60mg/kg/d i.p. + $AlCl_3$ 10mg/kg/d i.p. for 42d	*Nigella sativa*<br>Thymoquinone (TQ, purchased)<br>20 mg/kg/d p.o. during the last 14d of D-gal/$AlCl_3$.	MWM	TQ 20 mg/kg increased the time spent in the target quadrant in the MWM, vs. AD model group.
[99] Azzubaidi et al., 2012	Male SD rats	VD: 2-VO	*Nigella sativa*<br>NS oil from seeds (seeds purchased)<br>1 ml/kg p.o. for 10d prior to 2-VO and for further 70d after 2-VO	MWM	NS oil decreased the escape latency time and the total distance travelled, and increased the time spent in the target quadrant in the short- and long-term memory test group vs 2-VO, as well as in the working memory test vs. 2-VO model.
[100] Fanoudi et al., 2019	Male Wistar rats	VD: 2-VO	*Nigella sativa*<br>NS hydroalcoholic extract (NSE) from seeds, and thymoquinone (TQ, both (purchased)<br>NSE: 100, 200, and 400 mg/kg/d i.p. or TQ: 10, 20, and 40 mg/kg/d i.p. for 10d	MWM	NSE and TQ doses improved the escape latency and the pathway latency in a dose-dependent manner, in the MWM, vs. 2-VO model.<br>NSE 400 and TQ 40 mg/kg increased the time in the target quadrant in the MWM vs. the 2VO group.
[101] Abdelghany et al., 2022	Male SD rats	SCO-induced amnesia: SCO 2.2 mg/kg/d i.p. for 2 months before NS treatment	*Nigella sativa*<br>NS ethanolic extract from seeds (origins not specified)<br>400 mg/kg/d p.o. for 2 months	NORT<br>Y-maze<br>MWM	NS increased the difference score and the discrimination index in the NORT, and the percentage of the spontaneous alternation in the Y-maze vs. SCO model<br>Ns increased the time spent and the number of entries in the target quadrant, and decreased the first time to reach the platform in the MWM, vs. SCO model
[102] Poorgholam et al., 2018	Male Wistar rats	AD model: bilateral injection of 2 µL per side of Aβ1-42 1µg/µL	*Nigella sativa*<br>Thymoquinone (TQ, purchased)<br>5 and 10 mg/kg/d i.p. for 4 weeks	PA	TQ 10, but not 5 mg/kg, increased the initial latency and the step-through latency in the PA task vs. AD model group.
[109] Cheema et al., 2018	Male Wistar rats	Naïve	*Nigella sativa / Olea europaea*<br>NS oil from seeds (purchased)<br>OE 100% pure oil (purchased)<br>NS or OE 0.1 and 0.25 mL/kg/d p.o. for 5 weeks	EPM<br>MWM	NS 0.1 mL/kg increased the time the time spent in open arm after 1 wk, but reduced it after 4 wk of administration, in the EPM, vs. control.<br>OE 0.1 mL/kg increased the time the time spent in open arm after 5 wk, in the EPM, vs. control.<br>NS 0.25 mL/kg increased the time to reach the platform during training, while Ns 0.1 and 0.25 and OE 0.25 mL/kg increased the time to reach the platform at MWM test, vs control.
[111] Asghari et al., 2021	Male Wistar rats	STZ-induced diabetes model: STZ 60 mg/kg i.p.	*Olea europaea*<br>OE leaf extract (OLE, from a farm)<br>100, 200, and 400 mg/kg/d p.o. for 6 wk	PA	All the doses of OLE reverted to control levels the latency to enter in the dark chamber, and reduced the number of entries and the time spent less into the dark compartment in the PA, vs. control.
[113] Nitta et al., 1995	Male Wistar rats	SCO-induced amnesia: 0.075, 0.15, 0.3 mg/kg i.p.	*Panax ginseng*<br>Pg roots (purchased)<br>Ethanolic extract (EE), the water- (WSF) and the lipid-soluble fraction (LSF)<br>2, 4, 8 g/kg acute p.o.	8-ARM	SCO 0.3 impaired the performance in the 8-ARM<br>EE or WSF 8 g/kg, but not LSF, ameliorated the performance in the 8-ARM, vs control.
[114] Xu et al., 2016	Male ICR mice	SCO-induced amnesia: 3 mg/kg 15 min after WGOS	*Panax ginseng*<br>Pg roots (purchased)	MWM<br>NORT	WGOS 40 or 80 mg/kg reverted the impairment in the escape latency and in the number of platform crossing in the MWM, as well as the discrimination index in the NORT, vs. SCO model.

			Water-soluble ginseng oligosaccharides (WGOS): 40 and 80mg/kg i.p. for 4d before the tasks, and during the tasks		
[115] Hsieh et al., 2000	Male SD rats	SCO-induced amnesia: 1 mg/kg i.p.	*Panax ginseng* PG roots (purchased) 500 and 1000 mg/kg p.o. for 7d	PA	PG 500 and 1000 mg/kg reversed the reduction of the step-through latency at the PA retention test vs. SCO model.
[116] Al-Hazmi et al., 2015	Male SD rats	SCO-induced amnesia: 2 mg/kg i.p.	*Panax ginseng* PG root grain EtOH extract (purchased) 100 or 200 mg/kg/d p.o. for 14d	MWM	PG 100 and 200 mg/kg decreased the escape latency and increase the number of crossing over the platform position in the MWM, vs. SCO model.
[117] Ju et al., 2021	Male C57BL/6J mice Nrf2-WT and Nrf2-KO	SCO-induced amnesia: 1 mg/kg i.p.	*Panax ginseng* Red ginseng (RGE) and hydrolysed red ginseng extract (HRGE) (provided) RGE 300 mg/kg p.o. for 10d HRGE 50, 100 or 300 p.o. for 10d	Y-maze PA MWM	RGE 300 and HRGE 50, 100 and 300 mg/kg restored the spontaneous alternation in the Y-maze, the latency time in the PA and the escape latency in the MWM vs. SCO model in Nrf2-WT but not in Nrf2-KO mice.
[118] Huang et al., 2023	Male C57BL/6 mice	SCO-induced amnesia: 2 mg/kg i.p. for 2 weeks	*Panax ginseng* Pg roots with different cultivation procedures: in nutrient (D1), standard (D2) or smart farming (S2) conditions 200 mg/kg/d p.o. for 2 weeks.	NORT Y-maze PA	S2 condition at 200 mg/kg reverted to control levels the memory index of NORT, Y-maze and the step-through latency of the PA compared to D1 and D2 conditions.
[119] Pena et al., 2014	Male ICR mice	SCO-induced amnesia: 1 mg/kg i.p.	*Panax ginseng* Red ginseng (RG), Rg3, Ginseol k-g3 (purchased) RG: 100 mg/kg p.o. Rg3: 20 and 40 mg/kg p.o. k-g3: 12.5, 25, 50, 100, 200 mg/kg p.o. Dosing: every day before the tests	Y-maze PA MWM	RG, Rg3, and k-g3 did not influence performance in the Y-maze, vs. SCO model. All RG, Rg3 and k-g3 doses, except k-g3 12.5 mg/kg, increased the latency time in the PA retention test, vs. SCO model. k-g3 50 and 200 mg/kg reduced the escape latency during MWM training, at day 4 and 5 (50 mg/kg) and at day 5 (200 mg/kg), vs. SCO model. Rg3 40 and k-g3 50 and 200 mg/kg increased the swimming time in the target quadrant in the MWM, vs. SCO model.
[120] Lu et al., 2018	Male ICR mice	SCO-induced amnesia: 0.75 mg/kg/d i.p. for 9 days	*Panax ginseng* 20(S)-protopanaxatriol (PPT, purchased) 20 or 40 μmol/kg/d i.p. for 27 days	NORT MWM PA	PPT 40 reverted the deficits in the discrimination index in the NORT, vs. SCO model. PPT 20 and 40 decreased the escape latency on training day 4 and 5, and increased the number of crossing platform at test in the MWM, vs. SCO model. PPT 20 increased the latency to enter in, while 40 increased the time in the dark chamber in the PA, vs. SCO model.
[121] Peng et al., 2021	male ICR mice	D-gal induced dementia: 500 mg/kg/d i.p. for 9 weeks	*Panax ginseng* Panax ginseng and red ginseng (PG and RG, purchased) RG and PG: 800 mg/kg/d p.o. for 7 weeks	8-ARM	RG 800 mg/kg reduced the total time in the 8-ARM vs. D-gal model. RG 800 reduced the reference memory errors, while both PG and RG 800 mg/kg reduced the working memory errors in the 8-ARM compared to D-gal model.
[122] Li et al., 2016	Female Wistar rats	D-gal +$AlCl_3$ induced dementia: D-gal 60 mg/kg/d i.p. + $AlCl_3$ 40 mg/kg/d p.o. for 90d	*Panax ginseng* PG roots (purchased) Ginseng protein (GP) 50 or 100 mg/kg p.o. 2/day for the last 30d of AD induction	MWM	GP 50 and 100 mg/kg decreased the escape latency, and increased the number of crossing the platform and the time in the platform quadrant in the MWM, vs. AD model.
[123] Li et al., 2017	Female Wistar rats	D-gal +$AlCl_3$ induced dementia: D-gal 60 mg/kg/d i.p. + $AlCl_3$ 40 mg/kg/d p.o. for 90d	*Panax ginseng* PG roots (purchased) Ginseng protein (GP) 100 mg/kg p.o. 2/day for the last 30d of AD induction	MWM	GP 100 mg/kg decreased the escape latency, and increased the number of crossing the platform and the time in the platform quadrant in the MWM, vs. AD model.

[124]<br>Zhu et al.,<br>2014	Male SD rats	D-gal induced dementia:<br>D-gal 120 mg/kg/d s.c. for 42 days	*Panax ginseng*<br>Ginsenoside Rg1 (purchased)<br>20 mg/kg/d i.p. for 28d after 14d of D-gal	MWM	Rg1 decreased the latency to the platform during MWM training, vs. D-gal model.<br>Rg1 decreased the escape latency, and increased the number of platform crossing and the percentage of the target quadrant staying time in the MWM test, vs. D-gal model.
[125]<br>Chen et al.,<br>2018	C57BL/6 mice	D-gal induced dementia:<br>D-gal 120 mg/kg/d s.c. for 42 days	*Panax ginseng*<br>Ginsenoside Rg1 (purchased)<br>20 mg/kg/d i.p. for 28d after 14d of D-gal	MWM	Rg1 decreased the latency to the platform during MWM training, vs. D-gal model.<br>Rg1 decreased the escape latency, and increased the number of platform crossing and the percentage of the target quadrant staying time in the MWM test, vs. D-gal model
[126]<br>Zhong et al.,<br>2020	Male Kunming mice	D-gal +$AlCl_3$ induced dementia: D-gal 150 mg/kg s.c. + $AlCl_3$ 13 mg/kg p.o. for 42 days	*Panax ginseng*<br>Ginsenoside Rg1 (purchased)<br>10 or 20 mg/kg i.p. for 20 days	PA<br>MWM	Rg1 10 and 20 mg/kg decreased the number of avoidance errors in the PA, vs. model.<br>Rg1 10 and 20 mg/kg decreased the latency time during training, and increased the residence time and the distance moved at test in the MWM, vs. aging model.
[127]<br>Zhang et al.,<br>2022	male ICR mice	D-gal induced dementia:<br>D-gal 800 mg/kg i.p. for 8 weeks	*Panax ginseng*<br>Ginsenoside Rg2 (made in lab)<br>10 or 20 mg/kg/d p.o. for 4 weeks	MWM	Rg2 10 and 20 mg/kg increased the frequency of platform crossing and the percentage of time in the target quadrant in the MWM, vs. model.
[128]<br>Zhang et al.,<br>2019	Male Wistar rats	D-gal induced dementia:<br>D-gal 60 mg/kg/d i.p. for 60 days	*Panax ginseng*<br>Ginsenoside Rg3 (purchased)<br>20 mg/kg/d p.o. for 60 days with D-gal	MWM	Rg3 decreased the escape latency at training, and increased the crossing time and distance in the platform area at test in the MWM, vs. AD model.
[129]<br>Zhao et al.,<br>2009	Male SAMP8 mice	AD model: senescence-accelerated 4-m-o mice	*Panax ginseng*<br>Ginsenoside mixture (GIN, purchased)<br>50, 100 and 200 mg/kg/d p.o. for 7months	MWM<br>PA	GIN 100 and 200 decreased the escape latency at training and the time in the target quadrant and the number of platform crossing at test in the MWM, vs. model.<br>GIN increased the latency and decreased the error number in the PA, vs model.
[130]<br>Tan et al.,<br>2015	Male SD rats	AD model: advanced glycation end product (AGE) 500μg bilaterally in the CA3	*Panax ginseng*<br>PG root (purchased)<br>PG extract from roots (made in lab)<br>250, 500 and 1000 mg/kg p.o. for 30d	MWM<br>PA	PG 250, 500 and 1000 mg/kg decreased the latency to the platform at training, and 500 and 1000 increased the time in the target quadrant and the crossing time at test in the MWM.<br>PG 250, 500 and 1000 mg/kg reduced the number of errors and increased the latency in the PA, vs. model.
[131]<br>Yoon et al.,<br>2023	Male ICR mice	AD model:<br>AF64A 3 nmol/3 μL/mouse 0.5μL/min in right ventricle	*Panax ginseng*<br>Ginseng berry extract (GBE, made in lab)<br>100, 200 and 400 mg/kg/d p.o. for 6 wk	PA,<br>MWM	GBE 100, 200 and 400 mg/kg increased the retention time in the PA, vs. model.<br>GBE 100, 200 and 400 mg/kg decreased the escape latency in the MWM, vs. model.
[132]<br>Luo et al.,<br>2018	Male and female Wistar rats	AD model:<br>Aβ_{25-35} 2 μg/μL bilaterally in the CA1, 5 μL per area	*Panax ginseng*<br>PG roots (purchased)<br>PGL-1 (glycoproteins, extracted in lab)<br>40, 80 and 160 mg/kg i.p. for 35 days	MWM	PGL-1 80 and 160 mg/kg reduced the escape latency on day 3 and 4 at learning, and all the doses increased the frequency of crossing over the original platform at test in the MWM, vs model.
[133]<br>Cui et al.,<br>2021	Male SD rats	AD model:<br>Aβ25-35 1 μg/μL, 5 μL bilaterally in the Hipp	*Panax ginseng*<br>PG roots (purchased), Rg2 (made in lab)<br>25, 50 and 100 mg/kg/d p.o. for 6wk	MWM	Rg2 (all doses) decreased escape latency, and increased the number of platform crossing, of residence time in the platform area and the ratio between the distance travelled in the platform area and the total distance in the MWM, vs. model.
[134]<br>Song et al.,<br>2013	Male SD rats	AD model:<br>okadaic acid 40 ng/μL, 5 μL icv. in right ventricle	*Panax ginseng*<br>Ginsenoside Rg1 (purchased)<br>5, 10 or 20 mg/kg/d p.o. for 25d	MWM	Rg1, mainly 10 and 20 mg/kg, decreased the escape latency and increased the crossing of the platform frequency at training and test in the MWM, vs. model.
[135]<br>Chu et al.,<br>2014	Wistar rats (sex not specified)	AD model:<br>STZ 3 mg/kg, 10 μL 2 times icv in each lateral ventricle	*Panax ginseng*<br>Ginsenoside Rg5 (purchased)<br>5, 10 and 20 mg/kg p.o. for 27d	PA<br>MWM	Rg5 10 and 20 mg/kg increased the latency time in the PA, vs. model.<br>Rg5 5, 10 and 20 mg/kg increased the time in the target quadrant and the number of crossings in the MWM, vs. model.
[136]<br>Zhang et al.,<br>2014	Male SD rats	VD: MCAO	*Panax ginseng*<br>Ginsenoside Rd (purchased)<br>30 mg/kg i.p. 1h before MCAO and 10 mg/kg/d for 32d	MWM,<br>NORT	Rd increased the discrimination index at 1h and 24h retention test in NORT, vs. model.<br>Rd decreased the escape latency, and increased the path length, the percentage in the target quadrant, and the number of platform crossing in the MWM, vs model.

[137]<br>Wan et al., 2017	Male C57BL/6J mice	VD: 2-VO	*Panax ginseng*<br>Ginsenoside Rd (purchased)<br>10 or 30 mg/kg/d i.p. for 21d	MWM	Rd 10 and 30 mg/kg decreased escape latency and increased the time in the target quadrant, the distance in the target quadrant and the number of platform crossing in the MWM, vs. model.
[138]<br>Wang et al., 2023	Male SD rats	VD: 2-VO	*Panax ginseng*<br>20(S)-protopanaxadiol (PPD, purchased)<br>5, 10, 20 mg/kg/d s.c. for 3 weeks	MWM, Y-maze	PPD (all dases) decreased the time of escape latency on the 4th day and increased number of crossing platforms in the MWM, vs. control.<br>PPD 10 and 20 mg/kg increased the spontaneous alternation in the Y-maze, vs. control
[139]<br>Zhu et al., 2018	Male Wistar rats	VD: 2-VO + MCAO	*Panax ginseng*<br>Panax ginseng extract (PGE, purchased)<br>50 and 100 mg/kg p.o. for 8 wk	MWM	PGE 50 and 100 mg/kg decreased the escape latency time and increased percent distance in the target quadrant in the MWM, vs. model.
[140]<br>Nitta et al., 1995	Male Fischer 344 rats	Aging: 22 to 27-m-o rats	*Panax ginseng*<br>PG roots (purchased), EtOH extract<br>8 g/kg p.o. for 33d (8-ARM) or for 12d (operant discrimination task)	8-ARM<br>Operant discrimination task	PG decreased the number of errors and increased the number of successful rats in the 8-ARM, vs. control.<br>PG did not affect the discrimination learning in the operant discrimination task, vs. control.
[141]<br>Zhao, Li and Li, 2011	Female C57BL/6J mice	Aging: 12-m-o mice	*Panax ginseng*<br>Ginsenosides mixture (GIN, purchased)<br>0.028%, 0.056% and 0.112% w/v in drinking water for 8 months	MWM<br>PA	GIN did not affect the learning in the MWM, but increased the time spent in the target quadrant (except for 0.028%) and the number of platform crossing at test, vs control.<br>GIN 0.028, 0.056 and 0.112 % increased the latency and the error number in the PA test, vs control.
[142]<br>Lee, Oh 2015	Male C57BL/6 mice	Aging: 21-m-o mice	*Panax ginseng*<br>Red Ginseng (RG, purchased)<br>200mg/kg/d for 3 month	Y-maze<br>NORT<br>MWM	RG increased the spontaneous alternation index in the Y-maze, vs. control.<br>RG increased the exploration time of the novel object in the NORT, vs. control.<br>RG reduced the escape latency and increased the time spent in the target quadrant in the MWM, vs control.
[157]<br>Ahmed et al., 2014	Female APP transgenic mice	AD model: B6.129-Tg (APPSw) 40Btla/Mmjax; 24-30 months	*Punica granatum*<br>PG standard extract (Pomella, purchase)<br>100 and 200 mg/kg/d p.o. for 3 weeks	MWM<br>Y-maze	PG did not induce any effects at learning and test phase in the MWM, vs. control.<br>PG did not induce any effects in the spontaneous alternation ratio in the Y-maze, vs. control.
[158]<br>Akbarian et al., 2023	Male Wistar rats	SCO-induced amnesia: 2 mg/kg i.p. before behaviour	*Punica granatum*<br>PG (purchased), peel hydroalcoholic extract<br>200, 400 or 800 mg/kg/d p.o. for 3 weeks	MWM<br>PA	PG (all doses) decreased the travelling time and the distance travelled to find the platform at learning and increased the time and the distance travelled in the target area at test in the MWM, vs. SCO model.<br>PG (all doses) increased the latency to enter the black area and the time spent in the light area, while decreased the time in the dark and the entries in the black area at 3h, 48h and 72h post-shock in the PA, vs. SCO model.
[159]<br>Kumar et al., 2009	Male and female Swiss albino mice	Aging and SCO-induced amnesia: aging (14-m-o) or SCO (3-m-o with acute 1 mg/kg i.p.)	*Punica granatum*<br>PG seeds (purchased)<br>250 and 500 mg/kg/d p.o. for 21d	PA<br>EPM	PG 250 and 500 mg/kg increased the step-down latency in aged and young mice in the PA, vs. control.<br>PG 250 and 500 mg/kg decreased the transfer latency in aged and young mice in the EPM, vs. control.
[162]<br>Sreemantula et al., 2005	Male and female SD rats	SCO-induced amnesia: acute 1 mg/kg i.p.	*Vitis vinifera*<br>VV seed extract (fruit collected by lab)<br>100, 200 and 300 mg/kg p.o. for 7d	AA	VV dose-dependently ameliorated the learning of the AA task and the AA memory recovery after SCO-induced amnesia, vs. control.
[163]<br>Choi et al., 2023	Male C57BL/6 mice	SCO-induced amnesia: acute 0.8 mg/kg i.p. 30 min before each behavioural test	*Vitis vinifera*<br>Vitisin A (VA) purified from VV stembark (collected from vineyard)<br>1 or 100 ng/μL 3/wk in the third ventricle for 1 month	Y-maze<br>PA	VA 1 and 100 ng/μL increased the spontaneous alternation in the Y-maze test, vs. SCO model.<br>VA 1 and 100 ng/μL ameliorated the memory functions in the PA task, vs. SCO model.
[164]<br>Hong et al., 2021	Male C57BL/6J mice	SCO-induced amnesia: acute 0.8 mg/kg i.p. 30 min before each behavioral test	*Vitis vinifera*<br>Ampelopsin A (AA) purified from VV stembark (collected from vineyard)	NORT, PA	AA increased the novel object recognition index in the NORT test, vs. SCO model.<br>AA increased the step-through latency in the PA at test, vs. SCO model.

			10 ng/µL 0.5 µL, 3/wk into the third ventricle for 1 month		
[165] Rapaka et al., 2019	Male SD rats	AD model: $AlCl_3$ 100 mg/kg/d p.o. for 8 + 16 weeks	*Vitis vinifera*<br>VV fruits (purchased)<br>250 mg/kg and 500 mg/kg for 16 weeks with $ALCl_3$ treatment	MWM	VV 250 and 500 mg/kg decreased the escape latency during training and increased the time spent in the target quadrant at test in the MWM, vs. AD model.
[166] Lakshmi et al., 2014	Male SD rats	AD model: $AlCl_3$ 100 mg/kg/d p.o. for 45d	*Vitis vinifera*<br>Black grapes fruits (purchased)<br>Hydroalcoholic extract<br>400 mg/kg/d p.o. for 45d	PA	VV decreased acquisition time at training and increased retention time at test in the PA, vs. AD model.
[167] Borai et al., 2017	Male Wistar rats	AD model: $AlCl_3$ 17 mg/kg/d p.o. for 4 weeks	*Vitis vinifera*<br>VV leaves (collected in farm)<br>Leaves polyphenols extract (VLP)<br>100 mg/kg/d p.o. for 21d	T-maze	VLP decreased the time to reach the food and the time to achieve the task in T-maze, vs. AD model.

Abbreviations:

2-VO: common carotid arteries (2 vessels) occlusion; 4-VO: vertebral artery and common carotid arteries (4 vessels) occlusion; 8-ARM: 8-arm radial maze; AA: active avoidance task; AD: Alzheimer's Disease; $AlCl_3$: aluminium chloride; APP: amyloid precursor protein; Aβ: amyloid beta; Bkg: background; BMT: Barnes maze task; CA1: CA1 area of the hippocampus; d: days; D-gal: D-galactose; EPM: elevated plus maze; GB: Ginkgo biloba; Hcy: homocysteine; HHcy: Hyperhomocysteinemia; hipp: hippocampi; i.p.: intraperitoneal; icv: intracerebroventricular; MCAO: middle cerebral artery occlusion; MO: Melissa officinalis; m-o: months-old; MPTP: 1-methyl-4-phenyl-1,2,3,6-tetrahydropyridine; MWM: Morris water maze; NORT: novel object recognition task; NS: Nigella sativa; OE: Olea europaea; p.o.: per os; PA: passive avoidance tasks; PG: Panax ginseng; PG: Punica granatum; PS1: Presenilin 1; RoA: route of administration; s.c.: subcutaneous; SCO: scopolamine; SD: Sprague-Dawley; STZ: streptozotocin; Tg: transgenic; VD: vascular dementia; VV: Vitis vinifera; wk: week(s).
